# Side population cells derived from hUCMSCs and hPMSCs could inhibit the malignant behaviors of Tn^+^ colorectal cancer cells from modifying their *O*-glycosylation status

**DOI:** 10.1186/s13287-023-03334-3

**Published:** 2023-05-26

**Authors:** Wen Hu, Ruisong Ding, Mengyang Wang, Panpan Huang, Xia Wei, Xingyou Hu, Tao Hu

**Affiliations:** 1 Department of Immunology, Binzhou Medical University, Yantai, 264003 People’s Republic of China; 2 Qingdao University, Qingdao, 266071 People’s Republic of China

**Keywords:** Colorectal cancer cells, SP-hUCMSCs, SP-hPMSCs, *O*-glycosylation, Cosmc

## Abstract

**Background:**

Cosmc (C1GalT1C1) mutation could cause aberrant *O*-glycosylation and result in expression of Tn antigen on the surface of tumor cells (Tn^+^ cells), which is associated with the metastasis and prognosis of cancer progression. Mesenchymal stem cells (MSCs) could participate in immunoregulation, tissue damage repair, and tumor inhibition and be seen as an ideal candidate for tumor therapy due to their inherent capacity to migrate to tumor sites. However, their therapeutic effectiveness in different tumors is inconsistent and still controversial. Of note, emerging data reveal that side population (SP) cells have a stronger multilineage developmental potential than main population cells and can function as stem/progenitor cells. The effect of SP cells derived from MSCs on the biological behaviors and the *O*-glycosylation status of tumor cells remains unclear.

**Methods:**

SP cells were isolated from human umbilical cord MSCs (hUCMSCs) and human placenta MSCs (hPMSCs). Tn^+^ cells (LS174T-Tn^+^ and HT-29-Tn^+^ cells) and matching Tn^−^ cells (LS174T-Tn^−^ and HT-29-Tn^−^ cells) were isolated from human colorectal cancer cell (CRC) lines LS174T and HT-29 by immune magnetic beads. The proliferation, migration, apoptosis, Tn antigen expression, and *O*-glycome in Tn^+^ and Tn^−^ CRC cells before and after co-cultured with SP-MSCs were detected using real-time cell Analysis (RTCA), flow cytometry (FCM), and cellular *O*-glycome reporter/amplification (CORA), respectively. Cosmc protein and *O*-glycosyltransferase (T-synthase and C3GnT) activity in CRC cells were, respectively, assessed using western blotting and fluorescence method.

**Results:**

Both SP cells derived from hUCMSCs and hPMSCs could inhibit proliferation and migration, promote apoptosis of CRC cells, significantly reduce Tn antigen expression on Tn^+^ CRC cells, generate new core 1-, 2-, and 3-derived *O*-glycans, increase T-synthase and C3GnT activity, and elevate the levels of Cosmc and T-synthase protein.

**Conclusion:**

SP-hUCMSCs and SP-hPMSCs could inhibit proliferation and migration and promote apoptosis of Tn^+^ CRC cells via increasing *O*-glycosyltransferase activity to modify *O*-glycosylation status, which further adds a new dimension to the treatment of CRC.

**Supplementary Information:**

The online version contains supplementary material available at 10.1186/s13287-023-03334-3.

## Introduction

Colorectal cancer is one of the leading causes of cancer-related death [1]. Due to changes of life style and diet-related factors, morbidity is on the rise in recent years [2]. Traditional therapeutic methods such as surgical treatment, radiotherapy, and chemotherapy have the disadvantages of high recurrence rate and adverse reactions[3]. Furthermore, on account of the absence of early symptoms, many patients are diagnosed in the middle or advanced stages and often present local invasion and distant metastasis, which leads to a poor prognosis [4, 5]. With the development of cell therapy and related disciplines, mesenchymal stem cells (MSCs) have been widely used in the experimental and clinical studies of colorectal cancer, whereas the results are not encouraging. The reason is that MSCs can both promote and restrain tumor progression, and the effect of MSCs on different CRC cells is different, which may lie on several factors, such as the differentiation degree and source of MSCs, the difference between different tumor cells co-cultured with MSCs, and animal model of malignant tumor [6–8].

As far as mesenchymal stem cells (MSCs), they exist in almost all tissues and can be isolated from adipose tissue, bone marrow, umbilical cord, cord blood, and placenta [9]. They have the potential of self-renewal and multi-directional differentiation and, moreover, participate immune regulation, tissue damage repair, and tumor promotion or inhibition. Low immunogenicity, homing capacity, and chemotactic properties similar to immune cells that respond to injury and sites of inflammation are the important basis of MSCs for regenerative medicine and cancer therapy [10, 11]. Like most stem cells, they isolated from different tissues were consisting of various subpopulations and have certain heterogeneity which created variations in therapeutic efficacy. Among stem cells, side population cells (SP cells) have a high efflux activity and express a high concentration of efflux proteins on their surface membrane [12–14]. The degree of efflux activity is inversely correlated with their maturity, and such SP cells with the highest efflux activity are the most primitive or least restricted in the aspect of differentiation potential [15]. Notably, SP cells derived from normal tissues were different from tumor tissue and tumor cells. They derived from normal endometrium have multilineage developmental potential, while endometrial cancer cells show enhanced tumorigenicity [16]. Presumably, SP cells exist in MSCs derived from normal tissues, such as hUCMSCs and hPMSCs, may be different from their origin from cancer tissues, and could weaken the malignant biological properties of tumor cells.

The tumor microenvironment is a complex cell community including mutation cancer cells and non-neoplastic cells, even if the cells in the same tumor cell line may also express different surface markers and bear different phenotypes that result from abnormal *O*-glycosylation [17]. During the *O*-glycosylation, GalNAc linked to Ser/Thr formed GalNAc-1-*O*-Ser/Thr (Tn antigen) and further to be modified to complex structure Galβ1-3GalNAc-1-*O*-Ser/Thr (T antigen) by T-synthase [18]. Tn antigen can also be elongated into core 3 structures by Core 3 β1-3 *N*-acetylglucosaminyl-transferase (C3GnT) in colon tissue [19]. Due to T-synthase unique molecular chaperone Cosmc required for T-synthase bearing normal activity, its mutation could result in abnormal *O*-glycosylation and Tn antigen appearing on the surface of tumor cells (Tn^+^ cells) [20], while the expression of Tn antigen could influence cell proliferation, migration, and apoptosis [21]. In CRC, more than 70% (72–81%, 86.6% and 95%) of cases were Tn antigen positive [21–23]. IHC staining indicated that Tn antigen often appears in the apical cell membranes, mucin droplets, and the cytoplasm of most histological subsets of CRC tissues, including poorly differentiated adenocarcinomas and mucinous carcinomas [21]. Its expression was correlated with moderate histological differentiation and venous invasion of the tumors, metastatic potential, and poor prognosis [22–24]. If SP cells derived from MSCs could modify abnormal *O*-glycosylation, then they will change the biological behaviors of Tn^+^ tumor cells.

Based on these, we isolated SP cells from hUCMSCs and hPMSCs, as well as Tn^+^ (LS174T-Tn^+^ and HT-29-Tn^+^ cells with mutant Cosmc that have been identified in previous studies [25, 26]) and matching Tn^−^ cells (LS174T-Tn^−^ and HT-29-Tn^−^ cells with normal Cosmc) from colorectal cancer (CRC) cell lines (LS174T and HT-29) and established co-cultured system of SP-MSCs with CRC cells. The experiments showed that both of SP cells derived from hUCMSCs and hPMSCs could correct abnormal *O*-glycosylation in Tn^+^ CRC cells by increasing Cosmc and T-synthase protein, T-synthase, and C3GnT activity, and reducing the levels of Tn antigen expression on Tn^+^ cells. Meanwhile, restrain growth and migration, and enhance apoptosis of Tn^+^ and Tn^−^ cells.

## Materials and methods

### Cell culture

Human colorectal cancer (CRC) cell line LS174T (Procell CL-0145), HT-29 (CL-0118), and Human Umbilical Vein Endothelial Cells (HUVEC, CL-0675) were purchased from Procell Life Science and Technology Co., Ltd. (Wuhan, China). The cells were cultured in the high-glucose Dulbecco’s modified Eagle’s medium (DMEM, Hyclone) plus 10% fetal bovine serum (FBS, Gibco, Grand Island, NY, USA), and 100 U/mL penicillin–streptomycin (Solarbio, Beijing, China) at 37 °C and 5% CO_2_.

### Isolation and culture of hUCMSCs and hPMSCs

According to the previously described methods [9, 27], umbilical cord and placental tissue samples were obtained from healthy-term pregnant women from the Affiliated Yantai Hospital of Binzhou Medical College, Yantai, China, who signed informed consent forms. The project was approved by the institutional research ethics committee of Binzhou Medical College.

For the preparation of hUCMSCs, the umbilical cord samples were cut into 1 cm strips after removing their arteries and washed with phosphate-buffered saline (PBS) repeatedly. Then, the sample was homogenized in PBS into 1–2 mm^3^ fragments and placed in a petri dish with low-glucose DMEM supplemented with 10% fetal bovine serum after repeatedly rinsed with PBS. The Petri dishes were incubated at 37 °C in a 5% CO_2_, 95% humidity for 5 days, after which half of the medium was replaced with fresh medium every 3 days. Remove non-adherent tissue pieces and further culture for up to 10 days with medium change. When the cells reached 85–90% confluence, they were collected and amplified through passage culture.

For the preparation of hPMSCs, washed and crushed placental tissues were then incubated with collagenase IV (Gibco, Grand Island, USA) at 37 °C for 30 min. Low-glucose DMEM (Hyclone) supplemented with 10% fetal bovine serum (FBS, Gibco, Grand Island, USA) was used to terminate the collagenase-mediated digestion. Cell suspensions were collected after filtering out the large pieces of digestion tissue through a 100-mesh sieve and centrifuged at 800 g for 20 min. These cells were resuspended and adjusted to 1 × 10^5^ cells/mL with a complete culture medium (Low-glucose DMEM contained 10% FBS, 100 U/mL penicillin G, and 100 U/mL streptomycin sulfate). Then, cells were transferred to a 75 cm^2^ dish and statically cultured in an incubator at 37 °C and 5% CO_2_. The cell medium was exchanged either once or twice each week with a fresh medium.

Then, the phenotypic and morphological properties of isolated MSCs were identified by flow cytometry (FCM) and microscopy, respectively. Their multi-directional differentiation ability was analyzed by adipogenic and osteogenic staining methods.

### Isolation of SP cells derived from hUCMSCs and hPMSCs

hUCMSCs or hPMSCs cells were resuspended in Hoechst buffer and stained with Hoechst 33342 dye (Beyotime, Shanghai, China) for 90 min on a shaking platform at 37 °C. Cells were filtered through a 100 mm nylon membrane after being washed with PBS and then were analyzed and sorted using FACSAriaIII (BD Biosciences). The side population cells (SP-hUCMSCs and SP-hPMSCs) showed reduced fluorescence of both blue (670 nm) and red (450 nm) [12]. Their phenotypic and morphological properties and differentiation ability were identified as the same as above.

### Detection of stage-specific embryonic antigen-3 positive (SSEA-3^+^) cells

Cells (1 × 10^6^) were suspended in PBS and incubated with APC-labeled anti-SSEA-3 antibody (BioLegend, San Diego, CA, USA) for 60 min in the dark at 4 °C with gentle mixing every 10 min. After washing with PBS three times, the percentage of SSEA-3^+^ cells in hUCMSCs, hPMSCs, SP-hUCMSCs, and SP-hPMSCs were analyzed by flow cytometry.

### Cell sorting and Tn antigen detection

CRC cells (LS174T and HT-29 cells) were washed and resuspended with Buffer (PBS containing 0.5% FBS and 2 mM EDTA, PH 7.2) and labeled with primary mouse anti-Tn IgM mAb (provided by Dr. Tongzhong Ju at Emory University School of Medicine in Atlanta, GA, USA). According to the literature [28], Tn^−^and Tn^+^ cells in LS174T and HT-29 were sorted using immune magnetic beads, anti-mouse IgM MicroBeads (130–047-301, purchased from Miltenyi, Gladbach, Germany), and then were continuously cultured and used for subsequent experiments.

For detection of Tn antigen expression on the cell surface, 5 × 10^5^ cells were suspended in PBS and incubated with mouse anti-Tn IgM mAb for 1 h at 4 °C. Then, the cells were washed with PBS three times and incubated with APC-labeled goat anti-mouse IgM secondary antibody (Santa Cruz, CA, USA) for 0.5 h at 4 °C. After washing with PBS three times, the cells were analyzed by flow cytometry.

### The establishment of a co-culture system and isolation of CRC cells from the co-culture system

CRC cells (LS174T-Tn^−^, LS174T-Tn^+^, HT-29-Tn^−^, and HT-29-Tn^+^ cells) were labeled with CFDA SE (Beyotime, Shanghai, China) according to the manufacturer’s protocol and co-cultured with SP-hUCMSCs and SP-hPMSCs, which co-cultured with HUVECs were taken as control. In the co-culture system, each of four CRC cells (3 × 10^5^ cells) was mixed, respectively, with SP-hUCMSCs, SP-hPMSCs, and HUVECs (3 × 10^5^ cells) and loaded into 6-well plates at the density of 6 × 10^5^ cells/well. The mixed cells were cultured in the low-glucose DMEM plus 10% fetal bovine serum (FBS, Gibco, Grand Island, NY, USA), and 100 U/mL penicillin–streptomycin (Solarbio, Beijing, China) at 37 °C and 5% CO_2_ for 3 days. Then, cell suspensions were collected and analyzed using FACSAriaIII (BD Biosciences), of which the cells with green fluorescence were sorted and used for the subsequent experiments.

### Cell proliferation assay

CRC cells (LS174T-Tn^−^, LS174T-Tn^+^, HT-29-Tn^−^, and HT-29-Tn^+^ cells before and after co-cultured with SP-MSCs cells and HUVECs) were seeded in the E-plate (2 × l0^4^ cells/well in 100 μL of complete medium) and incubated at 37 °C and 5% CO_2_. Their proliferation was observed at 5 min intervals continuously from 0 to 72 h using a Real-Time Cell DP Analyzer (RTCA, xCELLigence, ACEA Biosciences, CA, USA). Cell index (CI) was recorded to reflect the alters of cell numbers at each time point. The rate of cell growth was computed according to the slope of the line between two given time points [29].

### Cell migration assay

RTCA was used to assess the migration of CRC cells. In brief, a complete medium was added to the lower chamber of the CIM plates (165 μL/well). DMEM without FBS was added to the upper chamber (30 μL/well). Then, CRC cell suspensions (LS174T-Tn^−^, LS174T-Tn^+^, HT-29-Tn^−^, and HT-29-Tn^+^ cells before and after co-cultured with SP-MSCs and HUVECs) were added into the upper chamber (3 × l0^4^ cells/well in 100 µL of DMEM without FBS) and incubated at 37 °C and 5% CO_2_ for 24 h. The values of cell index were recorded at 5 min intervals from 0 to 24 h and used to analyze the dynamic migration of cells in real time.

### Evaluation of cell apoptosis

The CRC cells (LS174T-Tn^−^, LS174T-Tn^+^, HT-29-Tn^−^, and HT-29-Tn^+^ cells before and after co-cultured with SP cells and HUVECs) were washed twice with PBS at 4 °C and suspended with binding buffer solution. Annexin V/PE and 7-AAD (Solarbio, Beijing, China) were added to cells and incubated for 15 min at room temperature away from light. Then, add PBS to the cells, and apoptosis was analyzed by FACS analysis using a flow cytometer (BD Biosciences, NJ, USA).

### Cytoplasmic protein extraction, T-synthase, and C3GnT activity assay

Cytosolic proteins were extracted from sorted co-cultured CRC cells using nuclear and cytoplasmic extraction reagents (Thermo Fisher Scientific, Waltham, MA, USA) according to the instructions, and their concentration was quantified using a bicinchoninic acid (BCA) protein assay kit (Solarbio, Beijing, China). T-synthase and C3GnT activity was measured using a fluorescent assay as described previously [28, 30].

### Western blotting

Cells were lysed with RIPA buffer containing 1 mM phenylmethylsulfonyl fluoride (PMSF, Solarbio Co., Ltd. Beijing, China) on ice for 30 min and then centrifuged at 16,000 × g for 20 min at 4 °C to obtain the cell extract. Protein concentration was determined with a BCA assay kit. 20 μg of protein was resolved using a 10% SDS-PAGE (Beyotime, Shanghai, China) Gel prior to being transferred to the PVDF membrane (Pall, NY, USA). The membranes were blocked with 5% BSA for 2 h and probed with respective primary antibodies overnight such as Rabbit anti-Cosmc (Abcam, England), Rabbit anti-T-synthase (Abcam, England), and anti-α-Tubulin (Proteintech, Rosemont, IL, USA) followed by incubating with appropriate secondary antibody conjugated with HRP (Santa Cruz Biotechnology, USA) at room temperature for 2 h. The interest proteins were detected by the ECL kit (Biosharp, Beijing, China). Blot images were captured using the chemiluminescence detection system (ChemisCope, UK). Protein values were normalized with loading control α-Tubulin.

### Detection of *O*-glycan by CORA

Profiles of *O*-glycan were detected by CORA as previously reported [31]. 3 × 10^5^ CRC cells sorted from before and after the co-cultured system were incubated with the compound Bn-α-GalNAc for 3 days and then collected the media to Sep-Pak C18 3 cc cartridge (Waters, Milford, MA, USA). After centrifugation Bn-*O*-glycans were purified and separated from flowthrough, eluted with organic solvents, freeze-dried, and permethylated. *O*-glycan structures were further analyzed by matrix-assisted laser desorption/ionization time-of-flight (MALDI-TOF) mass spectrometry (Agilent, Santa Clara, CA, USA).

### Statistical analyses

All experiments were repeated at least three times. The statistical analysis was performed using GraphPad Prism 8.0 and SPSS 23.0 software. Data were showed as mean ± standard deviation (mean ± SD). Student’s t tests were used to compare statistical differences between experimental groups. Categorical data were analyzed using the chi-square test. *P* < 0.05 was considered significant.

## Results

### Identification of MSCs and isolation of SP-MSCs

**Fig. 1:**
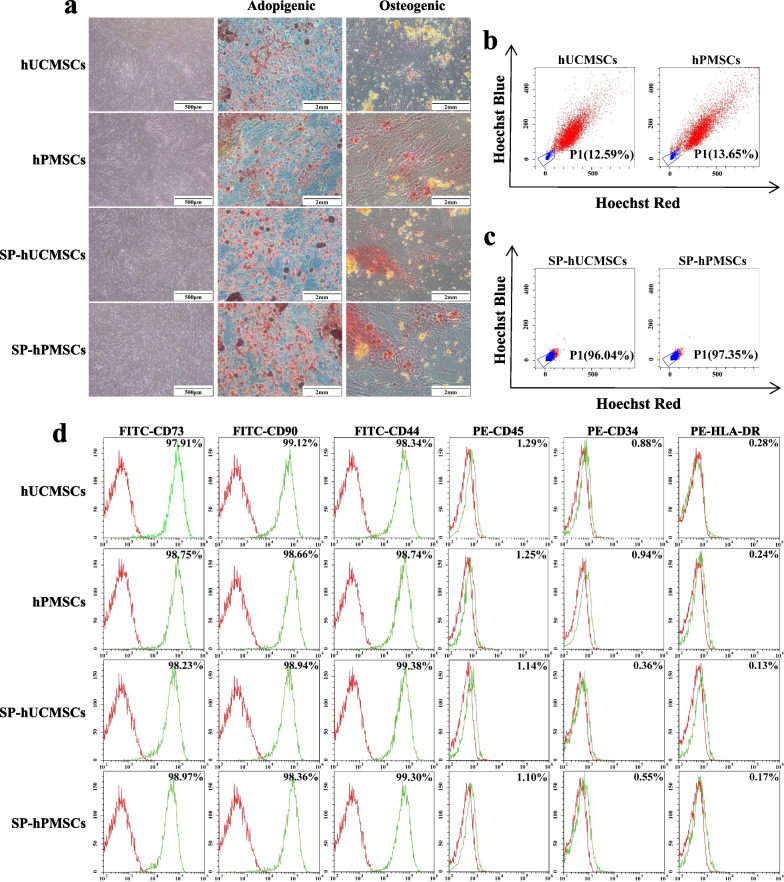
Identification of MSCs and SP-MSCs. **a** The morphology and differentiation of MSCs (hUCMSCs and hPMSCs) and SP-MSCs (SP-hUCMSCs SP-hPMSCs). MSCs and SP-MSCs showed typical fibroblastic morphology. Both of them could differentiate into adipocytes (positive for Oil Red O) and osteoblasts (positive for Alizarin Red), while SP-MSCs had stronger adipogenic and osteogenic differentiation ability. The image was captured with 1920*1440 resolution by OLYMPUS microscope and cellSens Entry. **b** The percentage of SP cells (P1) in hUCMSCs and hPMSCs. hUCMSCs and hPMSCs stained with Hoechst 33342 were analyzed using 350-nm excitation with blue (670 m) and red (450 m) emission. Among them, SP cells exhibited weak blue and red fluorescence. **c** The purity of sorted SP-hUCMSCs and SP-hPMSCs cells. All SP cells showed the reduction of both blue and red fluorescence. **d** Cell surface markers of MSCs and SP-MSCs were analyzed by FCM. The red histograms represented the isotype control, while green histograms represented specific marker expression on the cell surface

Microscopically, both of hUCMSCs and hPMSCs exhibited a typical fibroblastic morphology and could differentiate into adipocytes and osteoblasts when were cultured in adipogenic and osteogenic induction medium, which were identified using Oil Red O and Alizarin Red staining (Fig. 1a). Moreover, they expressed CD73, CD90, and CD44 but not CD34, CD45, and HLA-DR, which were consistent with the phenotypic characteristics of MSCs (Fig. 1d) [32]. From Hoechst 33342 staining, we found that there are approximate 12% and 13% SP cells in hUCMSCs and hPMSCs (Fig. 1b), respectively. They exhibited weak blue and red fluorescence due to the exclusion of the DNA-binding dye Hoechst 33342. Sorted these SP cells (SP-hUCMSCs and SP-hPMSCs) by FCM and then re-cultured for 72 h, their purity reached to about 96% and 97%, respectively (Fig. 1c). Their phenotypic characteristics have no changing compared with hUCMSC and hPMSCs, but they had stronger adipogenic and osteogenic differentiation ability (Fig. 1a, d).

### Identification of Muse cells in MSCs and SP-MSCs

Recently, a novel type of pluripotent stem cell with pluripotent marker stage-specific embryonic antigen-3 SSEA-3, multilineage-differentiating stress enduring (Muse) cells, was discovered in bone marrow, adipose tissue, dermis, and connective tissue, as well as cultured fibroblasts and MSCs [33]. They have self-renew and multi-directional differentiation potential and yet exhibit non-tumorigenic and low telomerase activities [34]. From detected the expression statues of SSEA-3 on hUCMSCs, hPMSCs, SP-hUCMSCs, and SP-hPMSCs, we discovered that the percentage of SSEA-3^+^ cells was approximately 1.17%, 1.22%, 1.23%, and 1.26%, respectively (Additional file 1: Fig. S1). The results promoted that the levels of muse cells have no significant difference in MSCs and SP-MSCs, and they are only a subpopulation of MSCs and SP-MSCs.

### SP-MSCs inhibit the expression of Tn antigen in Tn^+^ CRC cells and downgraded proliferation and migration of Tn^−^and Tn^+^ CRC cells

**Fig. 2:**
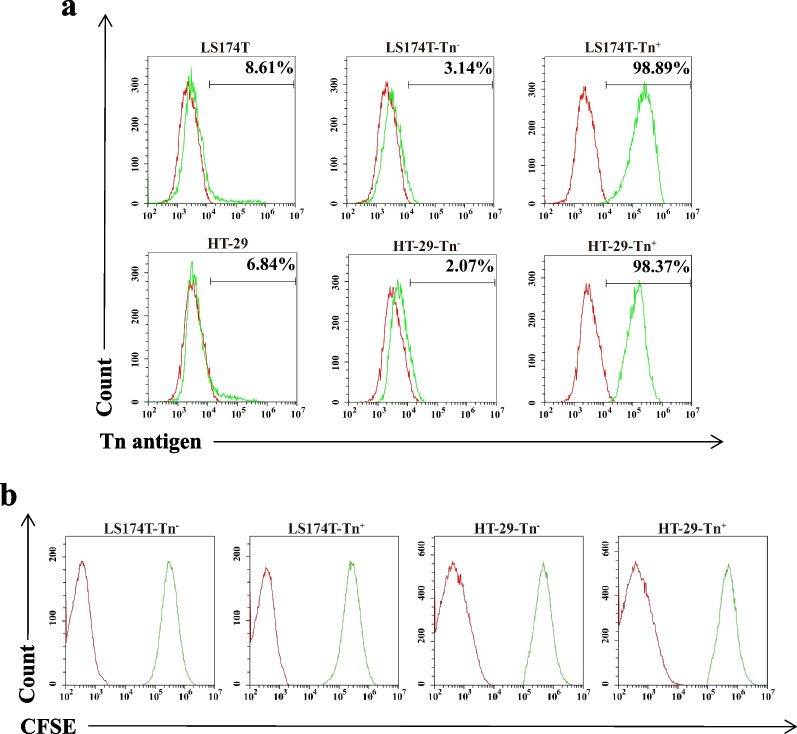
Sorting and labeling of CRC cells. **a** The percentage of Tn^+^ cells in LS174T and HT-29 cells before and after sorted by magnetic beads. **b** The labeling efficiency of LS174T-Tn^−^, LS174T-Tn^+^, HT-29-Tn^−^, and HT-29-Tn^+^ CRC cells with CFDA SE was detected by flow cytometry. Almost all CRC cells presented CFSE fluorescence. The red histograms represented the isotype control, while green histograms represented specific marker expression on the cell surface

The Tn antigen, as an *O*-glycoprotein with truncated *O*-glycans expressed on the cell surface, can influence cell–cell interactions and affect the biological behavior of cells and is strongly associated with many cancer progressions [35]. According to the Tn antigen expression, tumor cells were divided into Tn^−^ and Tn^+^ cells. Our previous study has shown that Tn^−^ and Tn^+^ cells coexisted in human CRC cell lines LS174T and HT-29 [25, 28]. Moreover, Tn^+^ cells such as Jurkat T and LS174T-Tn^+^ cells showed a stronger ability to proliferate and migrate, which could be downgraded by transfected Cosmc to inhibit Tn antigen expression [28]. Similar to these, FCM results showed that there were approximately 8.61% and 6.84% Tn^+^ cells in human CRC cell lines LS174T and HT-29 before isolation, respectively (Fig. 2a). After sorting using microbeads and re-cultured for 72 h, LS174T-Tn^+^ and HT-29-Tn^+^ cells reached to 98.89% and 98.37%, while LS174T-Tn^−^ and HT-29-Tn^−^ cells rise up to 96.86% and 97.93% and remaining tiny amounts of Tn^+^ cells, respectively (Fig. 2a). The proliferation and migration of LS174T-Tn^+^ and HT-29 Tn^+^ cells were higher than the corresponding LS174T-Tn^−^ and HT-29-Tn^−^ cells, respectively (Figs. 4a–c, 5a–c).

**Fig. 3:**
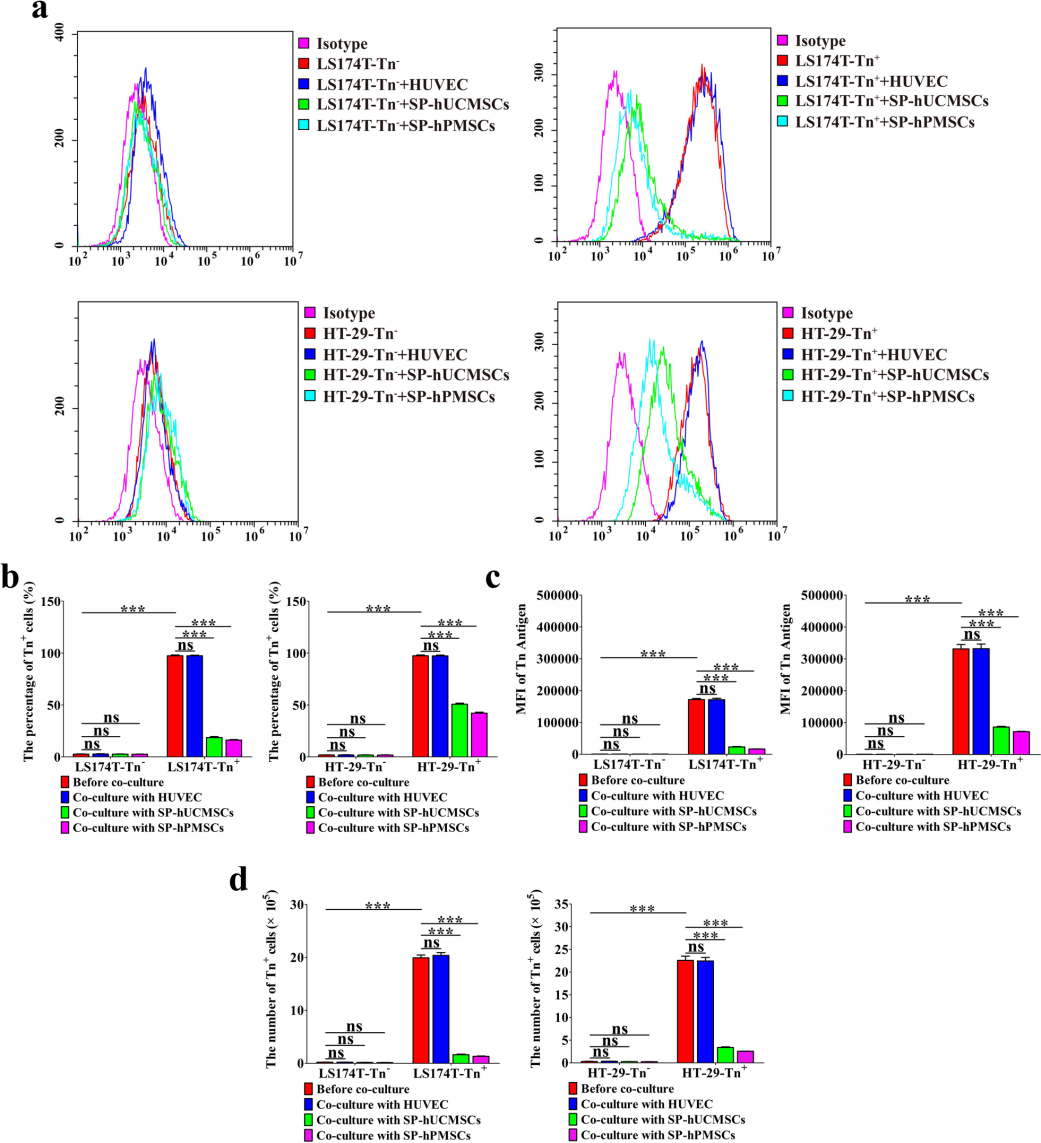
The expression of Tn antigen on CRC cells before and after co-cultured with SP-MSCs and HUVECs. **a** Tn antigen expression in LS174T-Tn^−^, LS174T-Tn^+^, HT-29-Tn^−^, and HT-29-Tn^+^ cells in different co-cultured systems was analyzed by FCM. SP-hUCMSCs and SP-hPMSCs but not HUEVCs could reduce even to abolish Tn antigen expression in LS174T-Tn^+^ and HT-29-Tn^+^ cells. **b** The percentage of Tn^+^ cells in LS174T-Tn^−^, LS174T-Tn^+^, HT-29-Tn^−^, and HT-29-Tn^+^ cells in different co-cultured systems. **c** The levels of Tn antigen represented by mean fluorescence intensive (MFI) in LS174T-Tn^−^, LS174T-Tn^+^, HT-29-Tn^−^, HT-29-Tn^+^ cells in different co-cultured systems. SP-hUCMSCs and SP-hPMSCs but not HUEVCs could reduce MFI of Tn antigen in LS174T-Tn^+^ and HT-29-Tn^+^ cells. **d** The absolute number of Tn^+^ cells in LS174T-Tn^−^, LS174T-Tn^+^, HT-29-Tn^−^, and HT-29-Tn^+^ cells in different co-cultured systems. Data shown are the mean ± SD of three independent experiments (*P < 0.05, **P < 0.01, and ***P < 0.001)

**Fig. 4:**
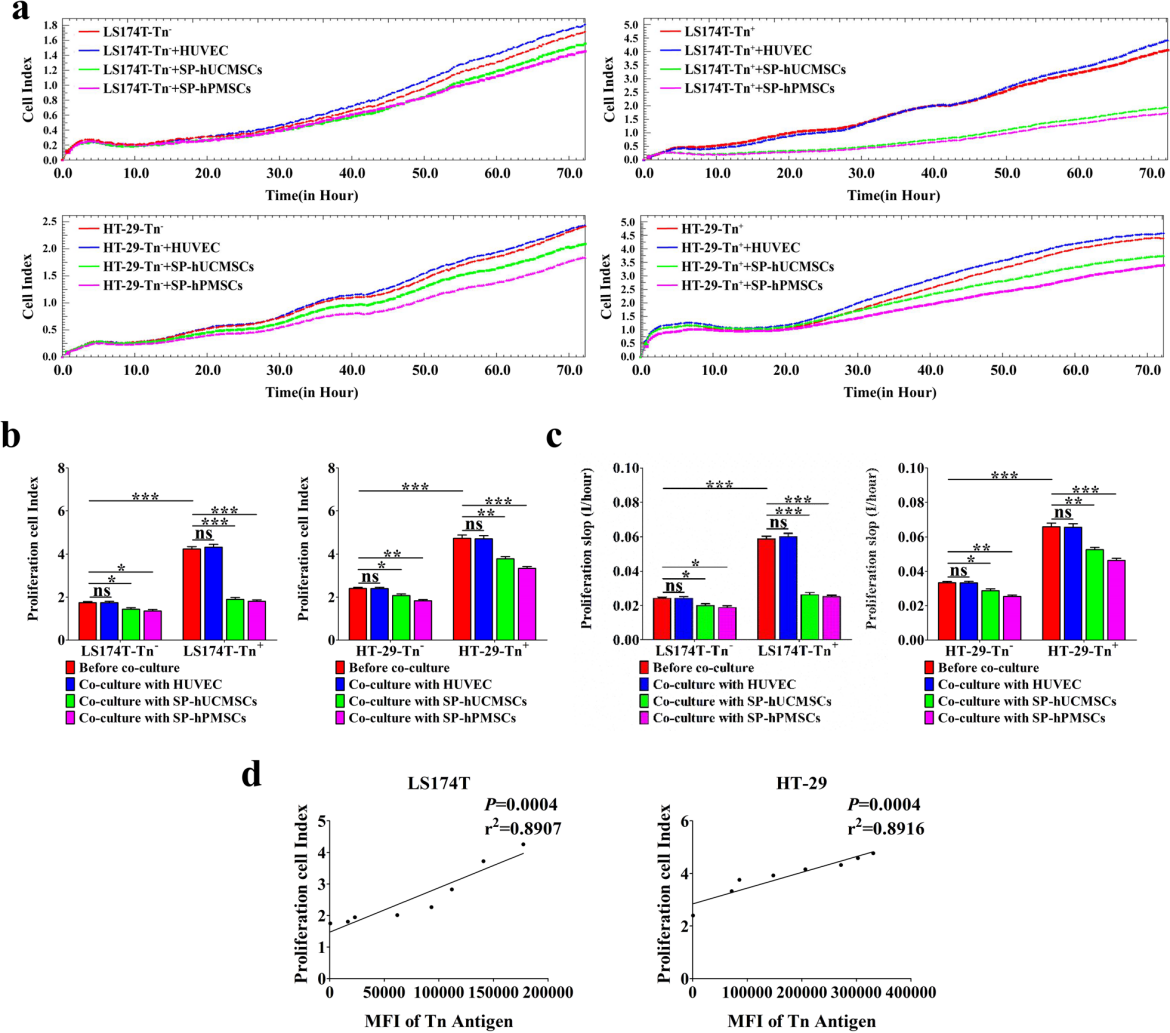
The proliferation of LS174T-Tn^−^, LS174T-Tn^+^, HT-29-Tn^−^, HT-29-Tn^+^ cells before and after cultured with SP-hUCMSCs, SP-hPMSCs, and HUVECs. **a** The proliferation curve of CRC cells in different co-cultured systems for 72 h was recorded by RTCA. **b** The variant of CRC cell index (CI) of proliferation at typical time points of 72 h. **c** The slopes for CRC cells proliferation at time points of 0–72 h. **d** The correlation of Tn antigen level (MFI) and proliferation CI of CRC cells. Data shown are the mean ± SD of three independent experiments (*P < 0.05, **P < 0.01, and ***P < 0.001)

**Fig. 5:**
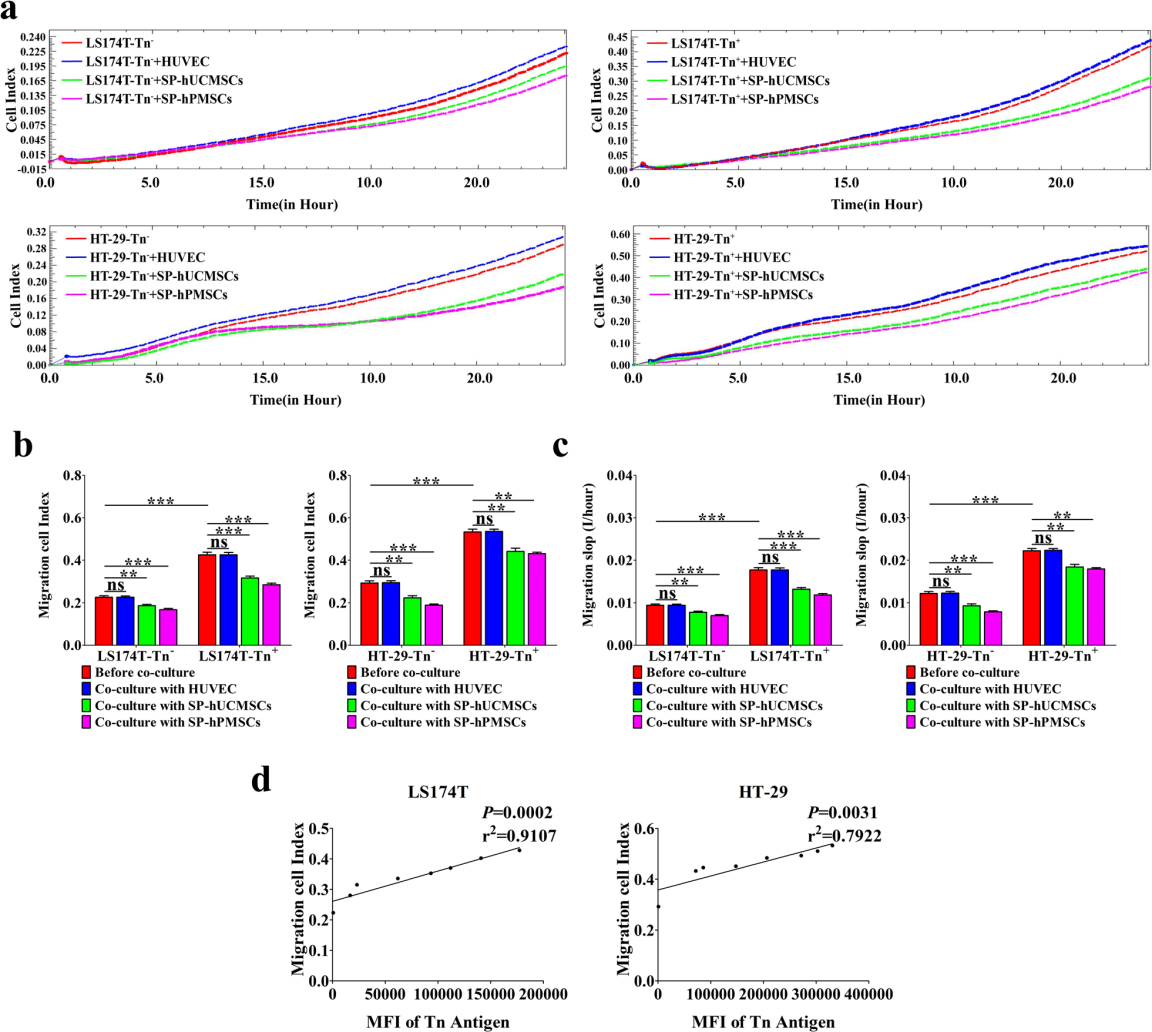
Migration of LS174T-Tn^−^, LS174T-Tn^+^, HT-29-Tn^−^, HT-29-Tn^+^ cells before and after cultured with SP-hUCMSCs, SP-hPMSCs, and HUVECs. **a** The migration curves of CRC cells in different co-cultured systems for 72 h were recorded by RTCA. **b** The variant of CRC cell index (CI) of migration at typical time points of 24 h. **c** The slopes for CRC cells migration at 0–24 h.** d** The correlation of Tn antigen level (MFI) and migration CI of CRC cells. Data shown are the mean ± SD of three independent experiments (*P < 0.05, **P < 0.01, and ***P < 0.001)

In order to better clear whether SP-MSCs affect the ability of proliferation and migration and Tn antigen expression of CRC cells, LS174T-Tn^−^, LS174T-Tn^+^, HT-29-Tn^−^, HT-29-Tn^+^ cells were labeled with CFDA SE (CFSE) before co-cultured with SP-MSCs (Fig. 2b), which contribute to distinguishing SP-MSCs and CRC cells in the co-cultured system. After co-cultured with SP-hUCMSCs, SP-hPMSCs, and HUVECs for 72 h, all cells were collected and stained with APC-labeled anti-Tn and were analyzed using FCM, in which those Tn^+^ CRC cells displayed both CFSE and APC fluorescence. Compared with prior co-culture, the percentage of LS174T-Tn^+^ cells reduced from 98.89 to 19% and 16% after co-cultured with SP-hUCMSCs and SP-hPMSCs, respectively, while HT-29-Tn^+^ cells dropped from 98.37 to approximately 50% and 42% (Fig. 3b). Moreover, the levels of Tn antigen represented by mean fluorescence intensity (MFI) were also obviously decreased in LS174T-Tn^+^ cells and HT-29-Tn^+^ cells (Fig. 3c). However, the percentage and absolute number of LS174T-Tn^+^ and HT-29-Tn^+^ cells, and their levels of Tn antigen have not significantly changed before and after co-cultured with HUVECs. The percentage and absolute number of LS174T-Tn^−^ and HT-29-Tn^−^ cells have also not significantly changed before and after being cultured with SP-hUCMSCs, SP-hPMSCs, and HUVECs (Fig. 3a, b, 3d). Furthermore, sorted CRC cells from the system co-cultured for 72 h, and then detected their proliferation and migration potency by RTCA. We observed that proliferation and migration of LS174T-Tn^−^, LS174T-Tn^+^, HT-29-Tn^−^, HT-29-Tn^+^ cells isolated from the systems of co-cultured with SP-hUCMSCs and SP-hPMSCs were all lower than that in prior co-cultured and control system of co-cultured with HUVECs (Figs. 4a–c, 5a–c). Moreover, the proliferation and migration of CRC cells were positive correlated with their Tn antigen level (Figs. 4d, 5d). These results indicated that SP-hUCMSCs and SP-hPMSCs but not HUEVCs could reduce even to abolish Tn antigen expression in LS174T-Tn^+^ and HT-29-Tn^+^ cells.

### SP-MSCs promoted apoptosis of Tn^+^ and Tn^−^ CRC cells

**Fig. 6:**
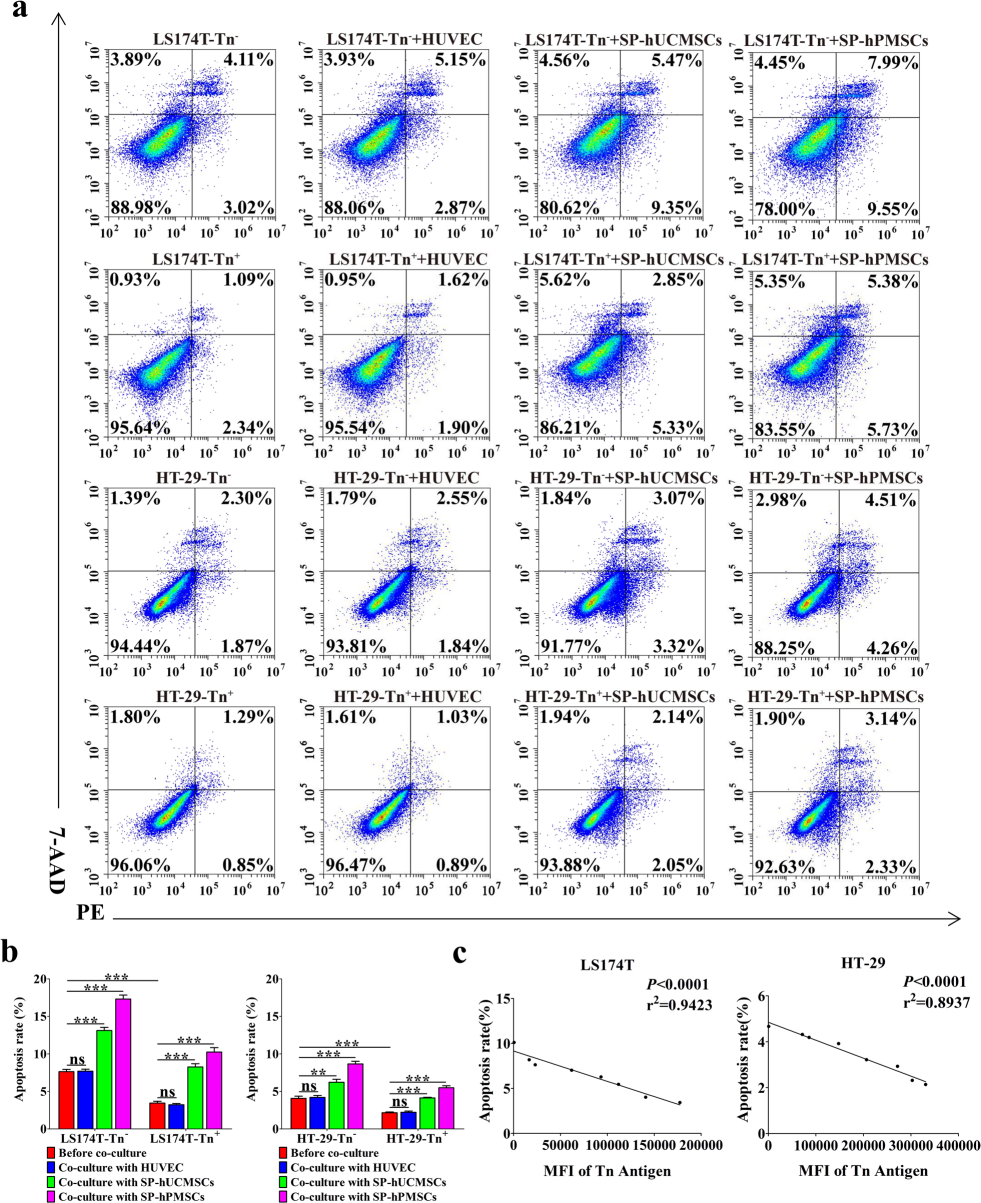
The effect of SP-MSCs on the apoptosis of CRC cells before and after co-cultured with SP-hUCMSCs, SP-hPMSCs, and HUVECs. **a** Representative images of FACS analysis for CRC cell apoptosis. **b** The changes of apoptosis rate of CRC. Cells in different co-cultured systems. **c** The correlation of Tn antigen level (MFI) and apoptosis rate of CRC cells. Data shown are the mean ± SD of three independent experiments (*P < 0.05, **P < 0.01, and ***P < 0.001)

Aberrant *O*-glycosylation resulted in Tn antigen expression on the tumor cell surface, which may inhibit apoptosis and provide tumor cell survival advantages through changing death receptor DR4 and/or DR5 activity. Our previous study has shown that Cosmc transfection could deduce Tn antigen expression on cell surface along with higher rates of apoptosis [28]. From detecting the apoptosis of CRC cells before and after co-culture with SP-MSCs and HUVECs using Annexin V-PE/7-AAD, we found that the rates of apoptosis of HT-29-Tn^+^ and LS174T-Tn^+^ cells before cultured with SP-hPMSCs, SP-hUCMSCs, and HUVECs were lower than that in the corresponding HT-29-Tn^−^ and LS174T-Tn^−^ cells (Fig. 6a, b). After co-cultured with SP-hPMSCs and SP-hUCMSCs, the apoptosis rate of all four CRC cells was higher than prior co-cultivation, but showed no significant changes before and after co-cultured with HUVECs (Fig. 6a, b). Moreover, the level of Tn antigen was negative correlation with apoptosis rate (Fig. 6c).

### SP-MSCs increased Cosmc and T-synthase protein, T-synthase and C3GnT activity in human CRC cells

**Fig. 7:**
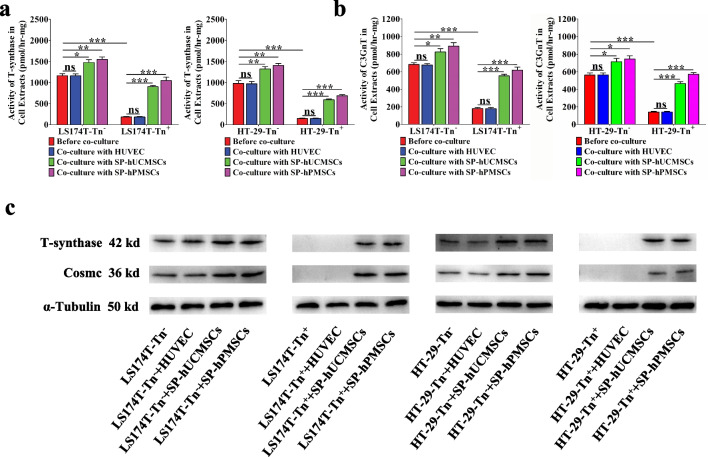
The changing of Cosmc and T-synthase protein, T-synthase, and C3GnT activity in CRC cells before and after co-cultured with SP-hUCMSCs, SP-hPMSCs, and HUVECs for 72 h. **a** The activity of T-synthase of LS174T-Tn^−^, LS174T-Tn^+^, HT-29-Tn^−^, HT-29-Tn^+^ cells before and after co-cultured with SP-hUCMSCs, SP-hPMSCs, and HUVECs was detected using a fluorescence method. **b** The C3GnT activity of LS174T-Tn^−^, LS174T-Tn^+^, HT-29-Tn^−^, HT-29-Tn^+^ cells before and after co-cultured with SP-hUCMSCs, SP-hPMSCs, and HUVECs was detected using a new fluorescence method. **c** Cosmc and T-synthase promote LS174T-Tn^−^, LS174T-Tn^+^, HT-29-Tn^−^, HT-29-Tn^+^ cells before and after co-cultured with SP-hUCMSCs, SP-hPMSCs, and HUVECs detected by Western blotting. Full length original blots are include in Additional file 1: Fig. S3. Three repeated Western blot experiments are include in Additional file 2.  Data shown are the mean ± SD of three independent experiments (*P < 0.05, **P < 0.01, and ***P < 0.001)

The extension of *O*-glycans depends on active T-synthase that transfers galactose (gal) from the donor (UDP-gal) to GalNAc to form core 1 structure *O*-glycans (gal β1-3 GalNAc-Ser/Thr, T antigen). Cosmc is vital for the right folding of T-synthase and bearing normal activity. In most cases, the exposure of Tn antigen is related to lower T-synthase activity and abnormal Cosmc protein [21]. Nonetheless, Core 3 β1-3 N-acetylglucosaminyl-transferase (C3GnT) transfers GlcNAc from UDP-GlcNAc to GalNAc to form the alternative core 3 structure *O*-glycans (GlcNAc-GalNAc-Ser/Thr), which appears to be limited to the gastric intestinal tract epithelia, maybe also mask Tn antigen. To determine the changes in T-synthase and C3GnT activity using fluorescent assay, we found that the T-synthase and C3GnT activity in Tn^+^ CRC (HT-29-Tn^+^ and LS174T-Tn^+^) cells was both lower than that in Tn^−^ CRC (HT-29-Tn^−^ and LS174T-Tn^−^) cells before co-cultured with SP-MSCs and HUVECs (Fig. 7a, b). Cosmc and T-synthase protein in Tn^+^ CRC (HT-29-Tn^+^ and LS174T-Tn^+^) cells was also lower than that in Tn^−^ CRC (HT-29-Tn^−^ and LS174T-Tn^−^) cells (Fig. 7c and Additional file 1: Fig. S3). However, T-synthase and C3GnT activity was both increased in all four CRC cells after co-cultured with SP-hPMSCs and SP-hUCMSCs, but showed no significant transformation when they were co-cultured with HUVECs (Fig. 7a, b). Moreover, T-synthase and Cosmc protein in Tn^+^ and Tn^−^ CRC cells after co-cultured with SP-hPMSCs and SP-hUCMSCs was increased, but has no changing in Tn^+^ and Tn^−^ CRC cells after co-cultured with HUVECs (Fig. 7c and Additional file 1: Fig. S3).

### SP-MSCs extended *O*-glycans in human CRC cells

**Fig. 8:**
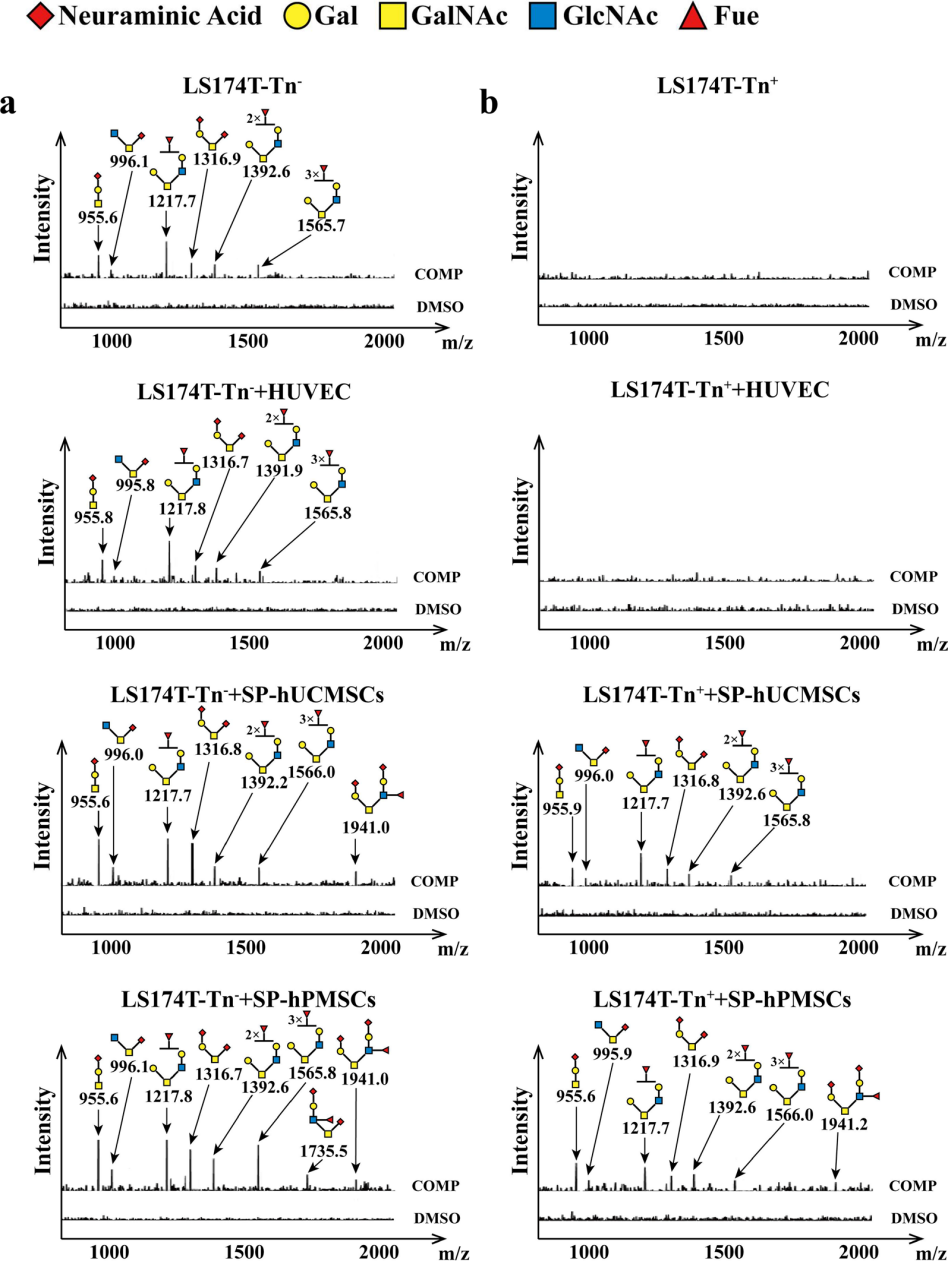
The profiles of glycans in LS174T cells detected by CORA in different co-cultured systems. The O-glycans in** a** LS174T-Tn^−^ cells, **b** LS174T-Tn^+^ cells before and after co-cultured with HUVECs, SP-hUCMSCs, SP-hPMSCs for 72 h, respectively

**Fig. 9:**
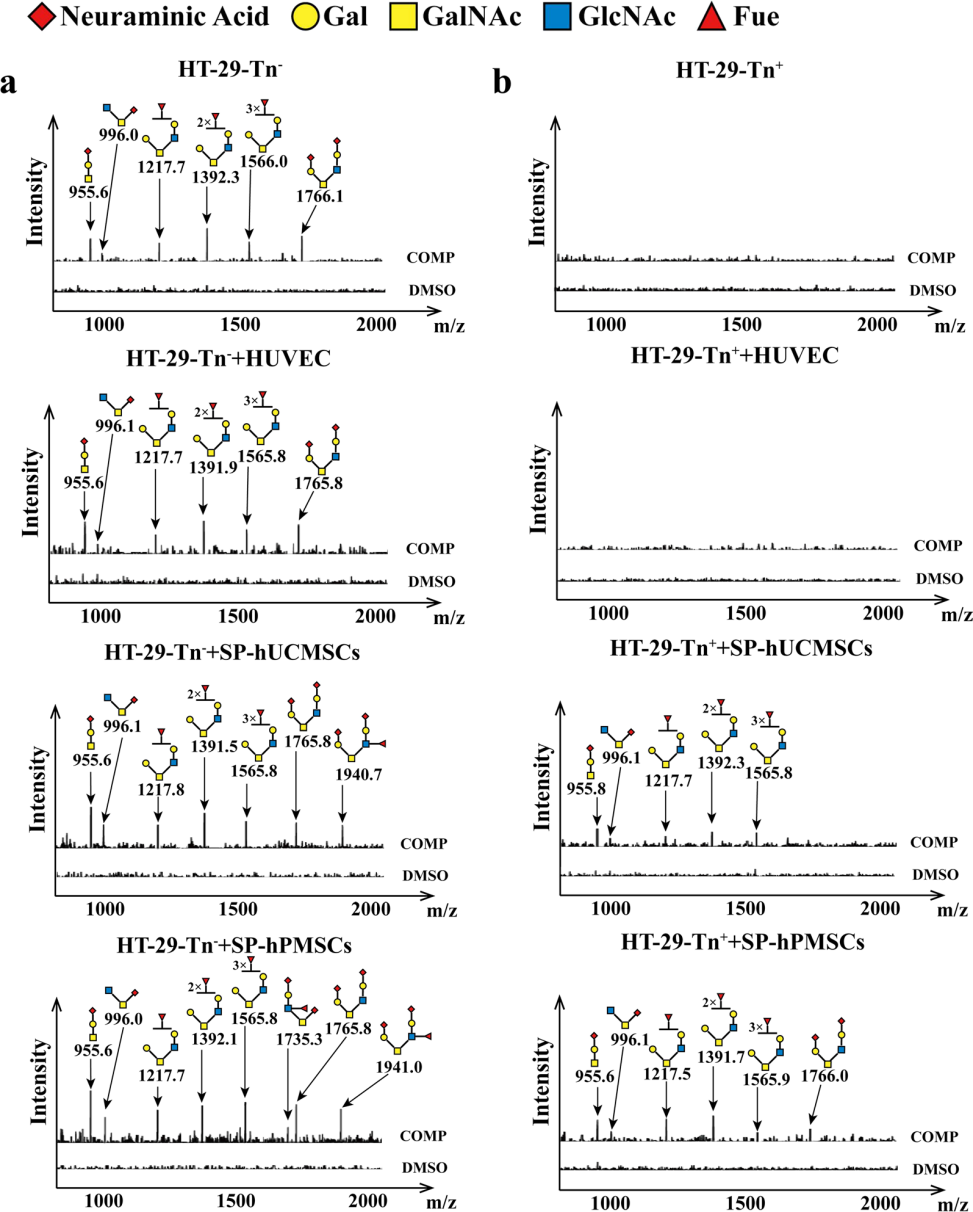
The profiles of O-glycans in HT-29 cells detected by CORA in different co-cultured systems. The O-glycans in **a** HT-29-Tn^−^ cells and **b** HT-29-Tn^+^ cells before and after co-cultured with HUVECs, SP-hUCMSCs, SP-hPMSCs for 72 h, respectively

T-synthase and Cosmc are essential to correct extension of core 1-derived *O*-glycans that play key roles in many biological processes, such as signal transduction, cell–cell interaction, immunity, and angiogenesis. By CORA, we found that Tn^−^ CRC (LS174T-Tn^−^ and HT-29-Tn^−^) cells existed in core 1-, core 2-, and core 3-derived *O*-glycans, while Tn^+^ CRC (LS174T-Tn^+^ and HT-29-Tn^+^) lack of core 1-, core 2-, and core 3-derived *O*-glycans (Figs. 8, 9 and Table 1). The components of *O*-glycans were the difference in LS174T-Tn^−^ and HT-29-Tn^−^ cells. LS174T-Tn^−^ presented core 1-derived *O*-glycans (m/z: 955.6, 1316.8), core 2-derived *O*-glycans (m/z: 1217.8, 1391.9, and 1566.0) based on core 1 structure, and core 3-derived *O*-glycans (m/z: 996.0) (Fig. 8a). HT-29-Tn^−^ cells emerged core 1-derived *O*-glycans (m/z: 955.6), core 2-derived *O*-glycans (m/z: 1217.8, 1391.9, 1566.0, and 1766.1), and core 3-derived *O*-glycans (m/z: 996.0) (Fig. 9a). These results indicate that the *O*-glycans in different CRC cells were not exactly the same. Tn^+^ CRC cells missing core 1, 2, and 3 structure *O*-glycans may be due to lower or the lack of T-synthase and C3GnT activity.

**Table 1:** Summary of O-glycan of CRCs in different co-cultured systems

O-glycans m/z	955.6	996.0	1217.8	1316.8	1391.9	1566.0	1735.5	1766.1	1940.2
LS174T-Tn^−^	**√**	**√**	**√**	**√**	**√**	**√**			
LS174T-Tn^−^ + HUVECs	**√**	**√**	**√**	**√**	**√**	**√**			
LS174T-Tn^−^ + SP-hUCMSCs	**√**	**√**	**√**	**√**	**√**	**√**			**√**
LS174T-Tn^−^ + SP-hPMSCs	**√**	**√**	**√**	**√**	**√**	**√**	**√**		**√**
LS174T-Tn^+^									
LS174T-Tn^+^ + HUVECs									
LS174T-Tn^+^ + SP-hUCMSCs	**√**	**√**	**√**	**√**	**√**	**√**			
LS174T-Tn^+^ + SP-hPMSCs	**√**	**√**	**√**	**√**	**√**	**√**			**√**
HT-29-Tn^−^	**√**	**√**	**√**		**√**	**√**		**√**	
HT-29-Tn^−^ + HUVECs	**√**	**√**	**√**		**√**	**√**		**√**	
HT-29-Tn^−^ + SP-hUCMSCs	**√**	**√**	**√**		**√**	**√**		**√**	**√**
HT-29-Tn^−^ + SP-hPMSCs	**√**	**√**	**√**		**√**	**√**	**√**	**√**	**√**
HT-29-Tn^+^									
HT29Tn^+^ + HUVECs									
HT-29-Tn^+^ + SP-hUCMSCs	**√**	**√**	**√**		**√**	**√**			
HT-29-Tn^+^ + SP-hPMSCs	**√**	**√**	**√**		**√**	**√**		**√**	

Of particular note is that the *O*-glycans have changed in CRC cells after co-cultured with SP-MSCs. A new core 2-derived *O*-glycan (m/z: 1940.2) was noted in LS174T-Tn^−^ and HT-29-Tn^−^ cells after co-cultured with SP-hUCMSCs and SP-hPMSCs, and a new core 3-derived *O*-glycan (m/z: 1735.5) appeared in LS174T-Tn^−^ and HT-29-Tn^−^ cells after co-cultured with SP-hPMSCs (Figs. 8a and 9a). Most importantly, six types of *O*-glycans (m/z: 955.6, 996.0, 1217.8, 1316.8, 1391.9, and 1566.0) and seven types of *O*-glycans (m/z: 955.6, 996.0, 1217.8, 1316.8, 1391.9, 1566.0, and 1940.2) appeared in LS174T-Tn^+^ cells after co-cultured with SP-hUCMSCs and SP-hPMSCs, respectively (Fig. 8b). Likewise, five types of *O*-glycans (m/z: 955.6, 996.0, 1217.8, 1391.9, and 1566.0) and six types of *O*-glycans (m/z: 955.6, 996.0, 1217.8, 1391.9, 1566.0, and 1766.1) appeared in HT-29-Tn^+^ cells after co-cultured with SP-hUCMSCs and SP-hPMSCs, respectively (Fig. 9b). Their components of *O*-glycans after co-cultured with SP-hUCMSCs were the same as the corresponding LS174T-Tn^−^ and HT-29-Tn^−^ cells without co-cultured, except for respective added a new *O*-glycan (m/z: 1940.2) (Figs. 8a, 9a). After co-cultured with SP-hPMSCs, LS174T-Tn^−^ and HT-29-Tn^−^ cells appeared two types of *O*-glycans (m/z: 1735.5,1940.2) (Figs. 8a, 9a). Even so, the *O*-glycans have no change in Tn^−^ and Tn^+^ CRC cells before and after being co-cultured with HUVECs (Figs. 8, 9). These results indicate that core 1, 2, and 3 structure *O*-glycans presented in Tn^+^ CRC cells after co-cultured with SP-MSCs may result from the increase of T-synthase and C3GnT activity.

## Discussion

MSCs were originally identified in the bone marrow and later discovered in many other tissues, and they could be recruited to tumor tissues and continue to exist in tumor tissue as T-MSCs. T-MSCs are not particularly prevalent in solid tumor tissues, accounting for only 0.01% of the total cell number within these tissues. Although the morphology and phenotype were similar to that MSCs isolated from normal tissue, their roles are different obviously [36, 37]. T-MSC supports tumor progression via secret immunosuppression factors to create a suitable microenvironment, and/or reprogramming by cancer cells in a tumor-specific manner [38, 39]. Thus, MSCs isolated from normal tissue, such as bone marrow, umbilical cord, placenta, endometrial polyps, menses blood, and adipose tissue, were used to treat a variety of diseases, including autoimmunity disease, tumor, and wound healing based on their ability to home to damaged tissues, multi-directional differentiation, and release of paracrine factors with pleiotropic effect [40]. The inherent capacity to migrate to tumor sites makes them becoming as valuable and potential attractive candidate for cancer therapy. However, the application of MSCs in cancer treatment has been impeded from contradictory results describing both promotion and suppressing of tumor effects in preclinical studies [40, 41]. The reason causing this controversy involved many kinds of factors, such as the source of MSCs, dose or timing of the MSCs therapy, type of cancer cells, and animal models of the malignant tumors. Obviously, from the point of source of MSCs, the proliferation and differentiation ability of bone marrow MSCs (BMSCs) displayed a great deal of variability due to the wide range in donor age and individual differences, which may influence their therapeutic efficacy [9, 42]. Although this variability has decreased in hUCMSCs and hPMSCs, it still could not change the facts that MSCs are a mixture of progenitors, in which SP cells bearing the highest efflux activity (i.e., have stronger bioactive mediators and exosomes releasing ability) and the most primitive differentiation properties maybe have an ideal efficacy for tumor treatment.

In this study, we discovered that SP-hUCMSCs and SP-hPMSCs all could inhibit proliferation and migration and promote apoptosis of Tn^+^ (LS174T-Tn^+^ and HT-29-Tn^+^) and Tn^−^ (LS174T-Tn^−^ and HT-29-Tn^−^) CRC cells (Figs. 4, 5, 6). The results are consistent with that engineering hPMSCs inhibit CRC progression and metastasis by inducing HT-29 cell death and suppressing proliferation [43], but different from that BMSCs promote HT-29 cell proliferation and invasion [44] and also support our hypothesis that SP-MSCs maybe could weaken malignant biological properties of CRC cells. These findings further suggest that SP-hUCMSCs and SP-hPMSCs could be used in CRC therapy, and the therapeutic effect may depend on their homing ability and the interaction of SP-MSCs and CRC cells in tumor sites. MSCs homing to tumor sites rested with the tumor microenvironment. Tumor is considered a wound that does not heal, and it produces an unremitting source of inflammatory mediators and leads to the infiltration of many inflammatory cells, which form an inflammatory microenvironment that attracts MSCs homing to tumor sites [45]. In colon cancer animal models, researchers use Rlu-eGFP (Renilla luciferase and enhanced GFP) gene transfected HT-29 cells and firefly luciferase (Fluc) gene transfected hPMSCs to track and detect tumor development and mesenchymal cell survival, respectively. The imaging signals showed that hPMSCs could migrate to the tumor sites [43]. Spatial transcriptomic analysis depicted infiltration of MSCs around Tn^+^ cells in CRC tissue (Additional file 1: Fig. S2). Thus, there is reason to suspect that SP-MSCs could also be homing to tumor sites. There are caveats, however, compared to MSCs homing and the effect of MSCs on tumor cells, and little research has addressed whether tumor cell affects on MSCs. Making clear the histological distribution of SP-MSCs and Tn^+^ cells, and that the characteristics of SP-MSCs interact with Tn^+^ cells in CRC tissues, will be an important future research direction.

Indeed, the source of MSCs could influence their anti-cancer activity, which was controlled by the mode of action of MSCs interacting with cancer cells including direct (cell-to-cell contact) and indirect (paracrine) contact. Direct and tight communication depended on membrane protein interactions and the swop of large plasma membrane fragments between MSCs and cancer cells, even extremely rare generated new cancer hybrid cells population through the fusion of these two cellular partners. Instead, indirect contacts are displayed by paracrine, and MSCs release soluble components such as cytokine, exosomes, and microRNAs affecting tumor cell cycle, cell proliferation, invasion, migration, apoptosis, angiogenesis, and/or adjusting the host immune response to tumor cells [46, 47]. Undoubtedly, direct and indirect action coexisted in the co-cultured system of MSCs with cancer cells, which is resulted in the complexity of the anticancer mechanism of different MSCs. For instance, BMSCs increased CRC KM12SM cells proliferation and migration and induce epithelial–mesenchymal transition (EMT) from up-regulation fibronectin by direct contact [46], umbilical cord-derived hUCMSCs reduced cell growth, and increased apoptosis in breast cancer by suppressing the activation of PI3K and AKT protein kinases [48], while perichondrium derived MSCs inhibit breast cancer cell growth through the DKK-1/Wnt/β-catenin signaling pathway [49]. Bone marrow-derived MSCs not only promote apoptosis and inhibit proliferation of glioma U251 cells via up-regulation of the IL-12 and IFN-γ levels and Bax/Bcl-2 expressions in tumor cells [50], but also restrict angiogenesis in ΔGli36 glioma xenograft via downregulation of the PDGF/PDGFR axis [51]. Human adipose tissue-derived MSCs suppressed melanoma A375SM and A375P cell proliferation by changing cell cycle distribution and inducing apoptosis in vitro and inhibited tumor growth in tumor-bearing nude mice by efficiently migrating to tumor tissues [52]. Most importantly, MSC-derived exosomes can entitle predominant signals to restrain neovascularization and to transmit the further tumor-inhibitory effects [53]. Moreover, healthy people BMSC-derived exosomes can inhibit in vitro angiogenesis in breast carcinoma cells through miR100 by regulating the mTOR/HIF1α/VEGF signal transduction pathway [54]. MSC-exosomal miR-3940-5p inhibits invasion, growth, metastasis, and EMT of CRC cells through targeting ITGA6 and the following TGF-β1 inactivation [55]. Therefore, releasing the ability of bioactive mediators and exosomes could determine or influence the effects of MSCs for tumor cells. In this study, we observed that T-synthase and C3GnT activity and Cosmc protein increased in CRC cells (LS174T-Tn^−^, LS174T-Tn^+^, HT-29-Tn^−^, and HT-29-Tn^+^cells) after co-cultured with SP-MSCs (SP-hUCMSCs and SP-hPMSCs) (Fig. 7) that appear along with abolishing or reducing of Tn antigen and generating new extended *O*-glycans (Figs. 3, 8, and 9). Maybe further to indicate that SP cells derived from hUCMSCs and hPMSCs exhibits the highest efflux activity and chemotactic exosomes characteristics could guarantee their secreted bioactive mediators and effectively penetrate into CRC cells. The effect of SP-MSCs on malignant biological properties of CRC cells may be from releasing T-synthase, C3GnT, and Cosmc protein to change their *O*-glycans.

For *O*-glycans, they play an essential role in many biological processes, such as signal transduction, cell–cell interaction, immunity, and angiogenesis [56]. Normal extension of *O*-glycans beyond Tn antigen, which is an intermediate of *O*-glycosylation exposure in the cell surface, has been regarded as arising from abnormal T-synthase, Cosmc, or C3GnT [17, 57]. T-synthase converts Tn antigen to Core 1-derived *O*-glycans, while C3GnT modifies Tn antigen to core 3-derived *O*-glycans. Both of them could cover Tn antigen expression on the cell surface. However, T-synthase bearing normal activity requires an endoplasmic reticulum (ER)‐resident molecular chaperone Cosmc which prevents the aggregation and subsequent proteasomal degradation of T-synthase [58]. Lack of Cosmc protein results in inactive T-synthase and aberrant *O*-glycosylation, characterized by the exposure of Tn antigen [59]. The present study demonstrated that the Tn antigen expression on Tn^+^ CRC cells was abolished and reduced after co-cultured with SP-MSCs (Fig. 3), and the levels of Cosmc protein significantly raised with increased activity of T-synthase and C3GnT (Fig. 7). Meanwhile, different types of extension of *O*-glycans include core 1-derived *O*-glycans (m/z: 955.6, 1316.8), core 2-derived *O*-glycans (m/z: 1217.8, 1391.9, 1566.0, 1766.1, 1940.2), and core 3-derived *O*-glycan (m/z: 996.0) appearing in Tn^+^ CRC cells (Figs. 8, 9). This means that SP-MSCs could recover the T-synthase and increased C3GnT activity coupled with the extension of *O*-glycans to reduce and mask Tn antigen expression in Tn^+^ CRC cells.

Usually, Tn antigen is considered as a tumor biomarker and strongly related to human colon cancer progression [60], which is expressed in human CRC cell lines HCT-116 and SW480 contributed to the reinforcement of migration and invasion by up-regulation of H-Ras expression that actives EMT process [35]. The inhibitor of glycosylation (benzyl-α-GalNAc) blocked oridonin combined with TRAIL (TNF-Related Apoptosis inducing Ligand, TRAIL)-induced apoptosis [61]. Masking Tn antigen via normal *O*-glycosylation could promote ligand-stimulated clustering of death receptor DR4 and DR5 and affected pro-apoptotic ligand Apo2L/TRAIL-induced apoptosis in tumor cells. Thus, altered *O*-glycosylation of tumor cells is a convenience for escaping from apoptotic signaling [62]. Beyond that, increased susceptibility to colitis and colorectal tumors in mice lacks core 3-derived *O*-glycans [57]. Human prostate cancer cells (PC3 and LNCaP cells) expressed high levels of core 3-derived *O*-glycans by transfection C3GnT cause to decreased cell invasion and migration, resulting in reduced prostate tumor formation and metastasis [63]. Moreover, our previous study discovered that wild-type Cosmc (WtCosmc) transfection could abolish Tn antigen expression, reduced proliferation, and migration and increased the sensitivity to apoptosis induced by Apo2L/TRAIL in LS174T-Tn^+^ cells, which correlated with WtCosmc transfection proper extension of *O*-glycans (core 1-derived *O*-lycans, m/z:955.6 and 1316.8) and restoration of mature *O*-glycosylation [28]. These further illustrate that intact *O*-glycans are necessary for cells to display normal biological characteristics.

Similarly, in the current study, we discovered that the SP-MSCs promote normal extension of *O*-glycans and increased the complexity of *O*-glycans in CRC cells, of which the core 1-derived *O*-glycan (m/z: 955.6, 1316.8) is helpful to apoptosis, and core 3-derived *O*-glycans (m/z: 996.0) are helpful to decrease cell invasion and migration, respectively. Both of them increased in Tn^−^ (LS174T-Tn^−^ and HT-29-Tn^−^) cells and newly generated in Tn^+^ (LS174T-Tn^+^ and HT-29-Tn^+^) cells after co-cultured with SP-MSCs (SP-hUCMSCs and SP-hPMSCs); further to reveal the mechanism of SP-MSCs decreased malignant biological properties of CRC cells from one side. In addition, our study also indicated that *O*-glycans in LS174T-Tn^−^ and HT-29-Tn^−^ cells were not exactly the same, except for existed five types of *O*-glycans (m/z: 955.6, 996.0, 1217.8, 1391.9, 1566.0), respective have core 1-derived *O*-glycan (m/z: 1316.7) and core 2-derived *O*-glycan (m/z: 1766.0), before co-cultured with SP-MSCs and HUVECs (Figs. 8a, 9a). Meanwhile, the newly generated *O*-glycans were also not exactly the same in CRC cells after co-cultured with SP-MSCs and HUVECs (Figs. 8, 9 and Table 1). These *O*-glycans with complicated structures decreased the levels of Tn antigen on the cell surface and increases the load for cell migration, and supporting our results that the level of Tn antigen is a positive correlation with proliferation and migration, and a negative correlation with apoptosis of CRC cells. Finally, it must be pointed out that there are two new subpopulation of cells among MSCs culture, such as pluripotent very small embryonic-like stem cells (VSEL) and muse cells, and were discovered and attracted researcher’s attention, recently. Due to VSEL cells (cell size 2–6 microns) being smaller than erythrocytes (about 7 microns) and other stem cells (over 10 microns), they maybe were not included in the MSCs and SP-MSCs when isolated by flow sorting. Moreover, the phenotypic features of VSEL cells are Lin^−^ CD34^+^CD45^−^ CD133^+^, and/or CXCR4^+^ [64, 65], while MSCs and SP-MSCs are CD34 negative (Fig. 1d). Thus, we think that MSCs and SP-MSCs were enrolled in current study maybe not VSELs. Otherwise, our results were also shown a small amount of Muse cells presented in MSCs (hUCMSCs and hPMSCs) and SP-MSCs (SP-hUCMSCs and SP-hPMSCs) (Additional file 1: Fig. S1), and their percentage has no difference in both cells, which means that Muse cells are a subpopulation for MSCs and SP-MSCs, respectively. Nonetheless, due to Muse cells being pluripotent but non-tumorigenic, and responsible for the triploblastic differentiation and tissue repair effects, moreover, they can proliferate rapidly if necessary and take part in more asymmetric divisions than self-renewal; their application to regenerative medicine and tumor therapy is highly anticipated [33, 34].

## Conclusion

In summary, our data showed that SP-hPMSCs and SP-hUCMSCs could inhibit oncogenic features of CRC cells such as proliferation and migration and promoted apoptosis by correcting their *O*-glycosylation status by increasing Cosmc and T-synthase protein, T-synthase, and C3GnT activity to abolish and reduce the expression of Tn antigen, which further adds a new dimension to the treatment of CRC.

## Supplementary Information

**Additional file 1:** Supplementary figures.

**Additional file 2:** Three repeated Western blot experiments.

## Data Availability

All data generated or analyzed during this study are included in this published article.

## Abbreviations

MSCs: Mesenchymal stem cells
hUCMSCs: Human umbilical cord mesenchymal stem cells
Muse cells: Multilineage-differentiating stress enduring cells
hPMSCs: Human placenta mesenchymal stem cells
CRC: Colorectal cancer cells
RTCA: Real-time cell analysis
FCM: Flow cytometry
CORA: Cellular *O*-glycome reporter/amplification
SP cells: Side population cells
C3GnT: Core 3 *β*1-3*N*-acetylglucosaminyl-transferase
HUVEC: Human umbilical vein endothelial cells
PBS: Phosphate-buffered saline
