# Supplementary material for: Side population cells derived from hUCMSCs and hPMSCs could inhibit the malignant behaviors of Tn+ colorectal cancer cells from modifying their O-glycosylation status
Source: Stem Cell Res Ther. 2023 May 26;14:145. doi: 10.1186/s13287-023-03334-3 (PMC10224610; doi:10.1186/s13287-023-03334-3)
Supplement: Supplementary file 1 — Additional file 1: Supplementary figures. [file 13287_2023_3334_MOESM1_ESM.doc]

**Supplementary**

**Detection of stage-specific embryonic antigen-3 positive (SSEA-3^+^) cells.**

Cells (1×10^6^) were suspended in PBS and incubated with APC-labeled anti-SSEA-3 antibody (BioLegend, San Diego, CA, USA) for 60 min in the dark at 4°C with gentle mixing every 10 min. After washing with PBS three times, the percentage of SSEA-3^+^ cells in hUCMSCs, hPMSCs, SP-hUCMSCs and SP-hPMSCs were analyzed by flow cytometry.


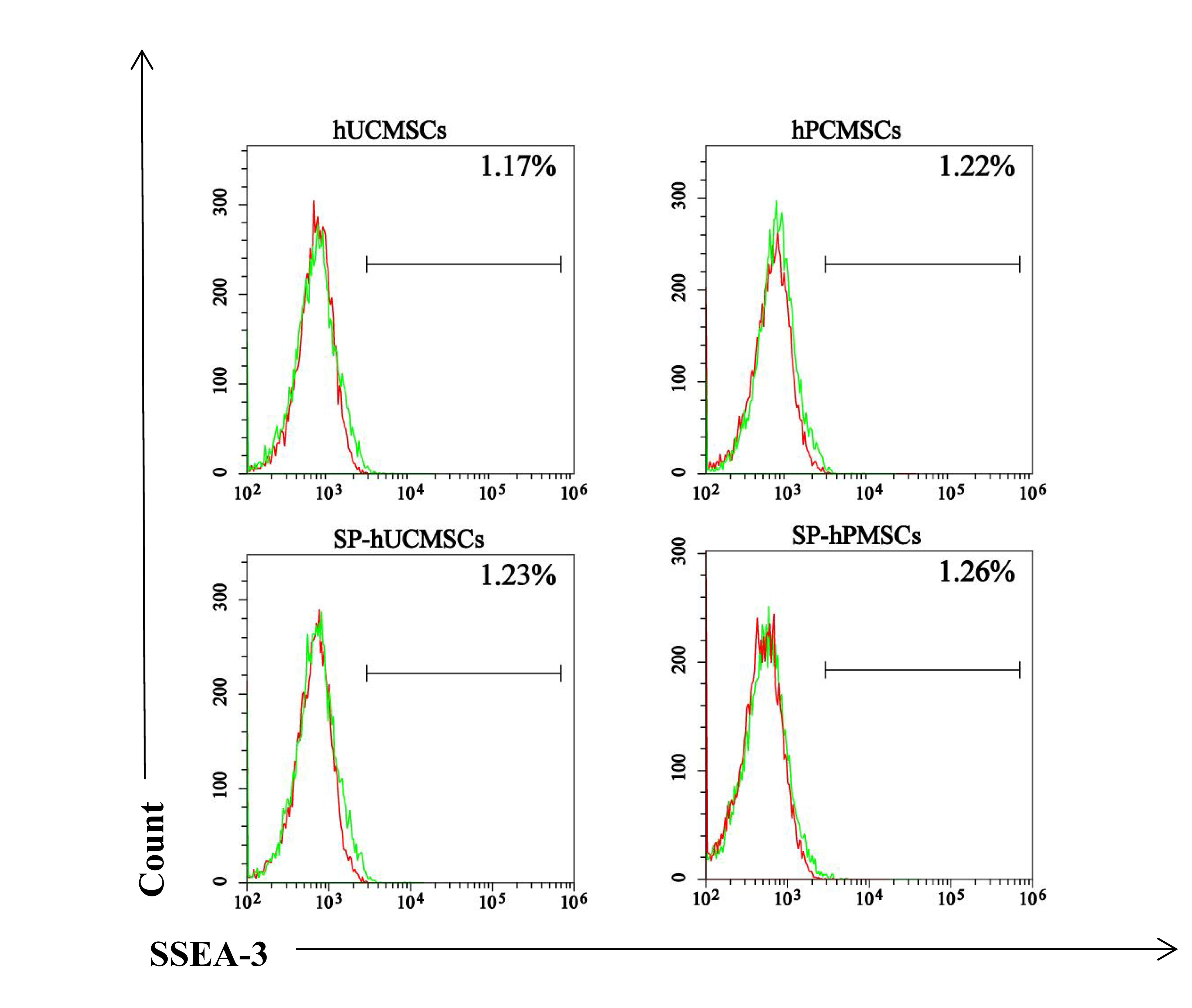


**Suppl. Fig. 1** SSEA-3 expression in hUCMSCs, hPMSCs, SP-hUCMSCs and SP-hPMSCs were analyzed by FCM. The red histograms represented the isotype control, while green histograms represented specific marker expression on the cell surface.

**Spatial transcriptomic analysis depicted infiltration of MSCs around Tn^+^ cells in CRC tissue**

To date, the interaction of MSCs with Tn^+^ cells in CRC tissues has not been reported, which may be due to MSCs with multiple markers (CD73^+^CD90^+^CD44^+^ CD34^-^CD45^-^HLA-DR^-^). The varied markers of MSCs from various organs lead to the difficulties in labelling MSCs on histological sections. In this study, following your insightful comments, we supplemented investigation based on spatial transcriptome (Suppl. Fig. 2). However, there is no direct coding gene for Tn antigen, and thus, the Tn^+^ cells were identified as C1GALT1C1^-^ B3GNT6^-^ cells. Meanwhile, the MSCs were primarily defined as CD73^+^CD90^+^CD44^+^ GD2^+^CD34^-^CD45^-^HLA-DR^-^ cells. As the results, MSCs were detected around Tn^+^ cells.


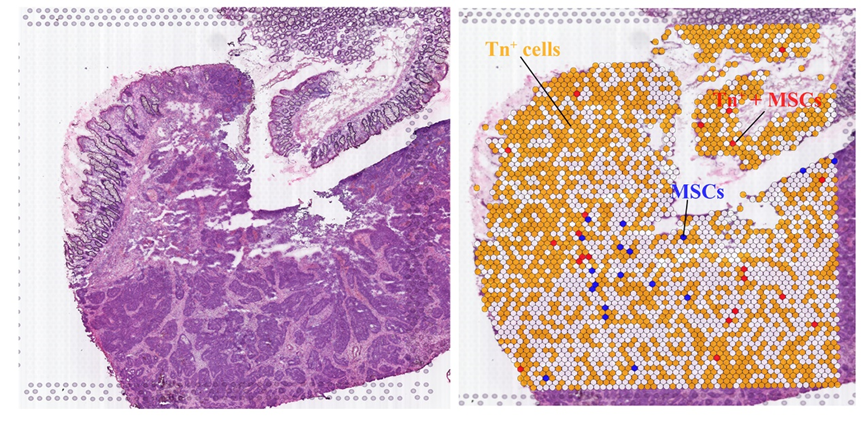


**Suppl. Fig. 2** Spatial relationships between MSCs and Tn^+^ cells illustrated by spatial transcriptomic analysis. HE staining of CRC tissue (left), Spatial plot of Tn^+^ cells and MSCs (right). The raw data was downloaded from the dataset The National Omics Data Encyclopedia (<https://www.biosino.org/> node/project/detail/OEP001756, and the data process was carried out according to the original document [1]. Here, the presence of Tn^+^ cells (orange point) and MSCs (blue point) were identified as C1GALT1C1^-^ B3GNT6^-^ cells and CD73^+^CD90^+^CD44^+^ GD2^+^CD34^-^CD45^-^HLA-DR^-^ cells, respectively. The red point represented concurrence of Tn^+^ cells and MSCs.

Reference

[1] Wu, Y. et al. Spatiotemporal Immune Landscape of Colorectal Cancer Liver Metastasis at Single-Cell Level. Cancer Discov 12, 134-153


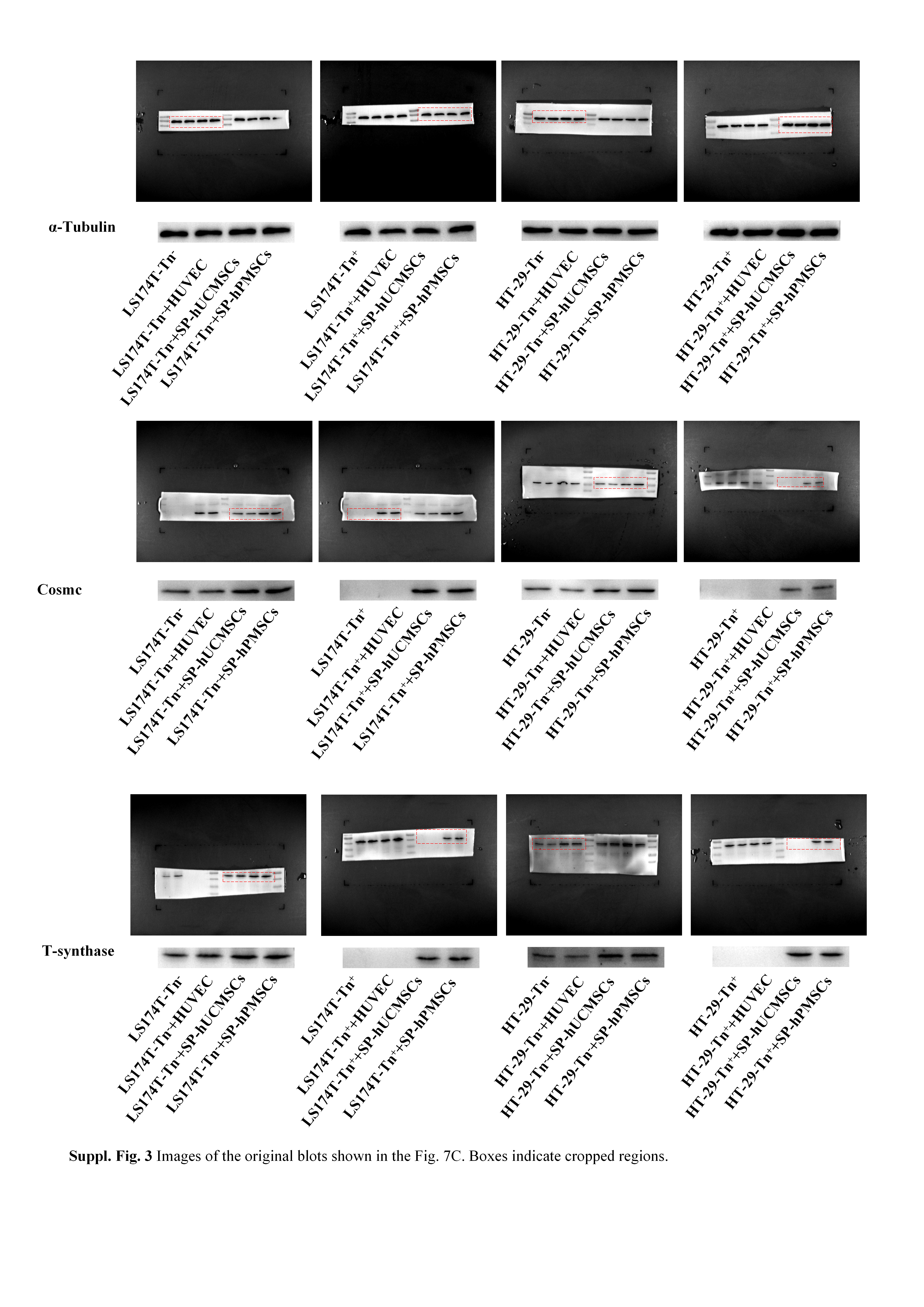


**Suppl. Fig. 3** Images of the original blots shown in the Fig. 7C. Boxes indicate cropped regions.
