# Supplementary material for: Side population cells derived from hUCMSCs and hPMSCs could inhibit the malignant behaviors of Tn+ colorectal cancer cells from modifying their O-glycosylation status
Source: Stem Cell Res Ther. 2023 May 26;14:145. doi: 10.1186/s13287-023-03334-3 (PMC10224610; doi:10.1186/s13287-023-03334-3)

# WB repeated experiments 1

## α-Tubulin

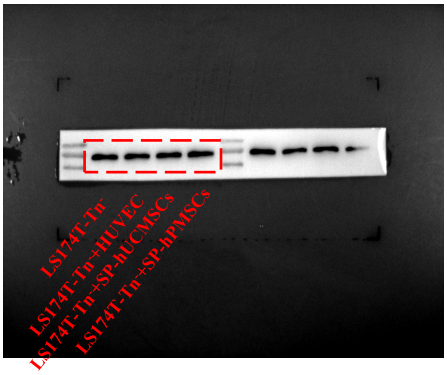


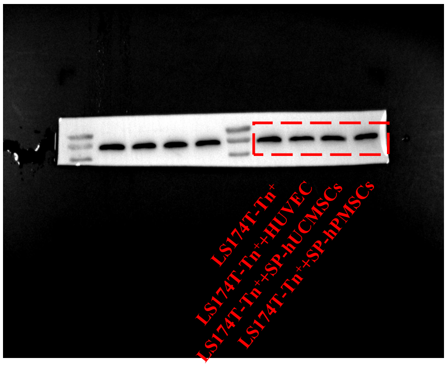


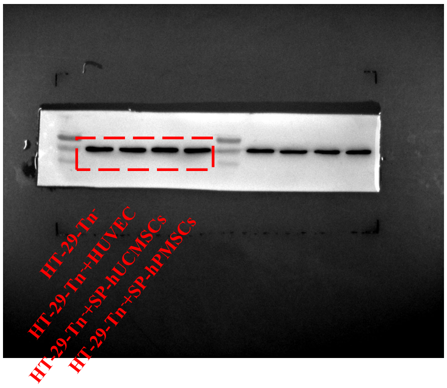


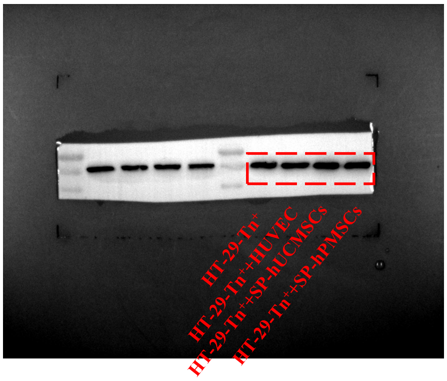


## Cosmc

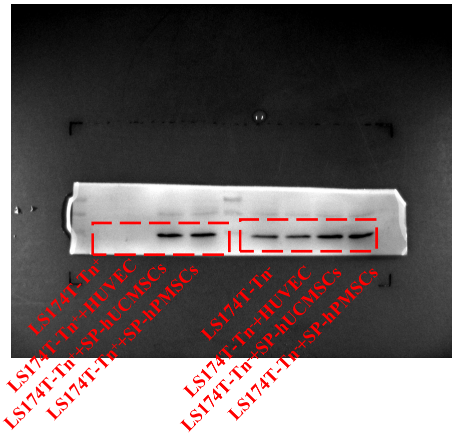


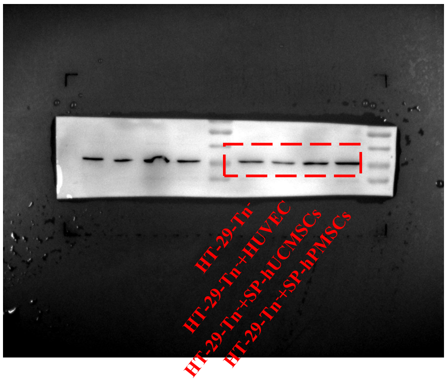


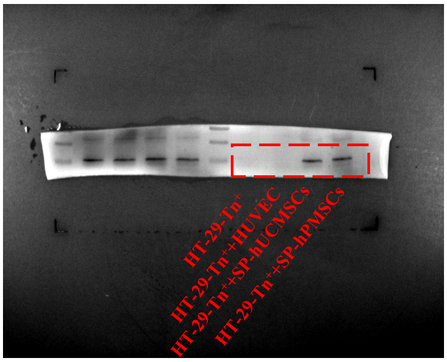

WB repeated experiments 1

**T-synthase**

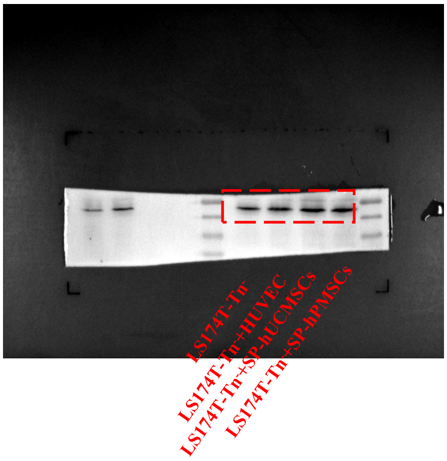


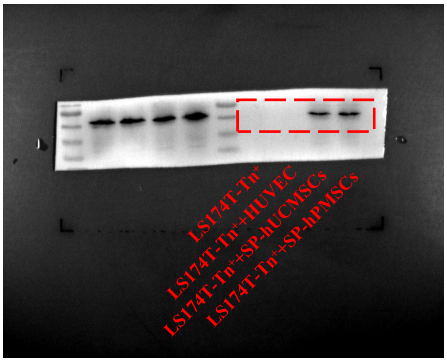


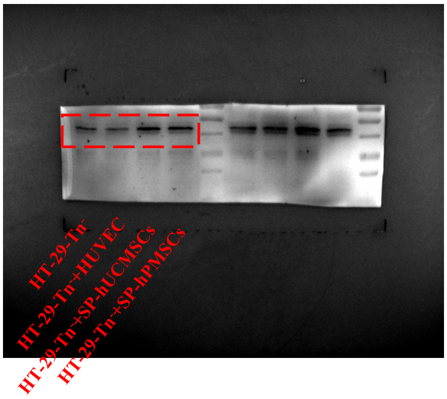


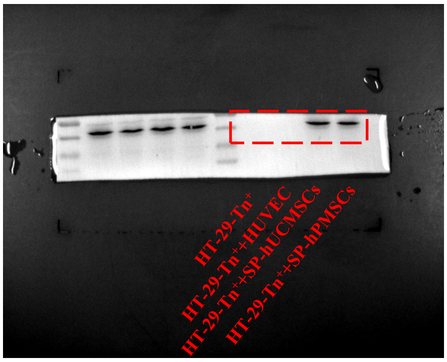


Blots in the Fig. 7 are from WB repeated experiments 1. Boxes indicate cropped regions.

# WB repeated experiments 2

## α-Tubulin

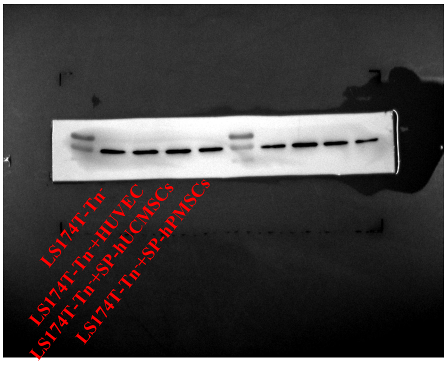


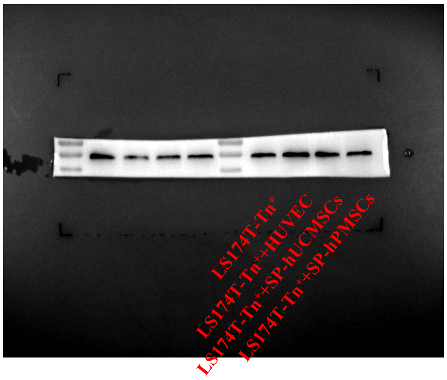


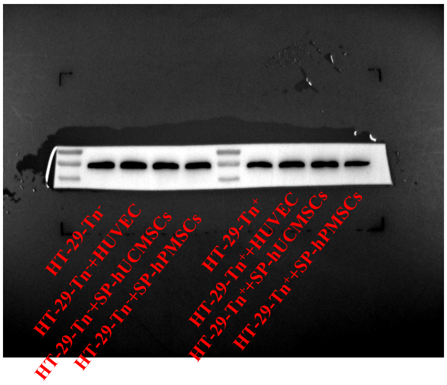


## Cosmc

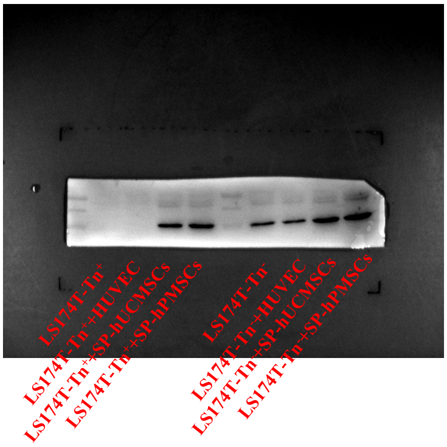


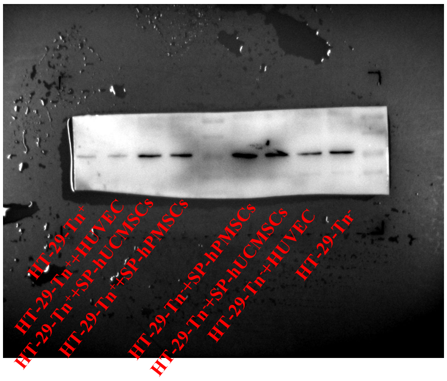


## T-synthase

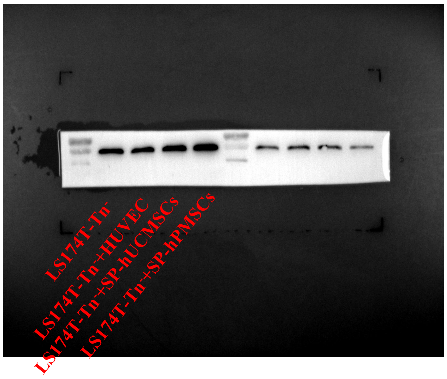


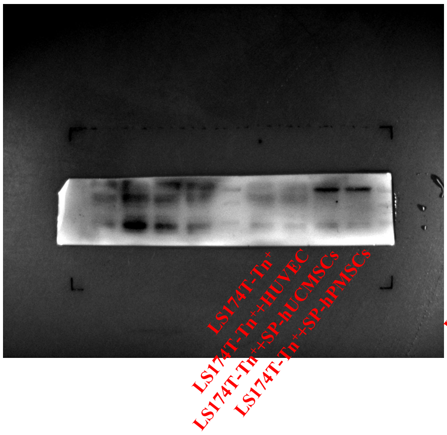


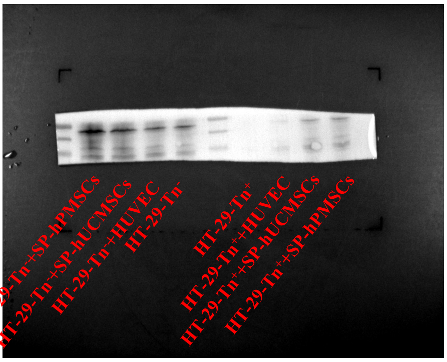

# WB repeated experiments 3

## α-Tubulin

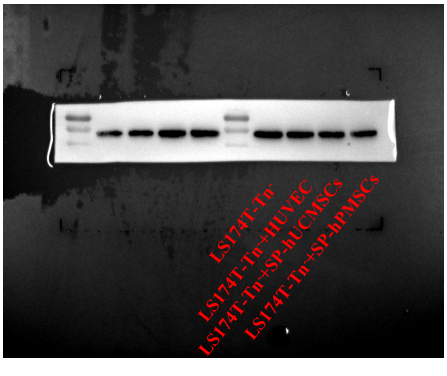


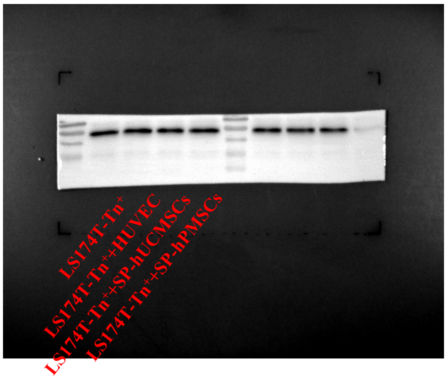


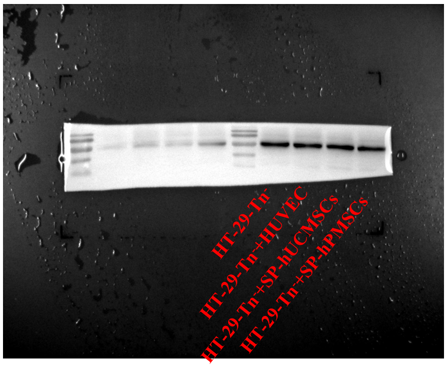


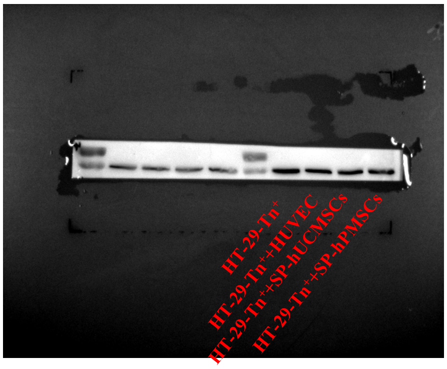


## Cosmc

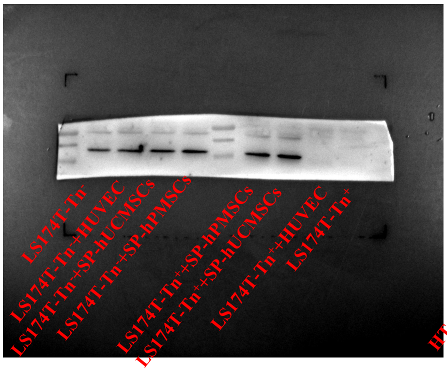


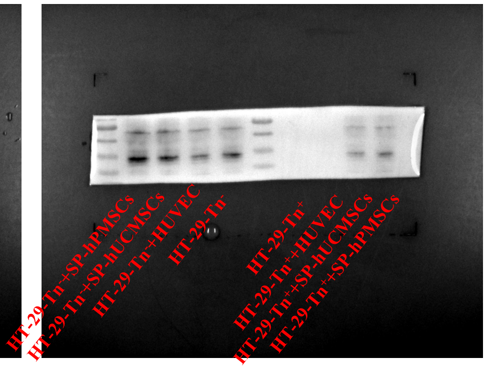

# WB repeated experiments 3

## T-synthase

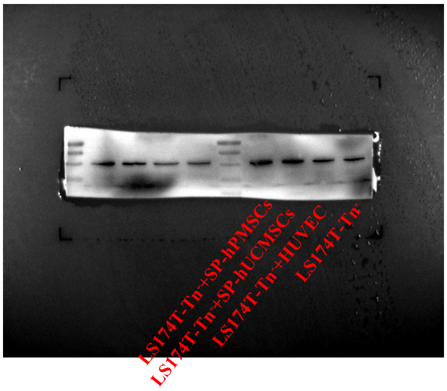


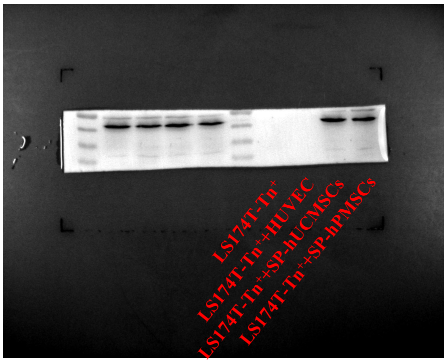


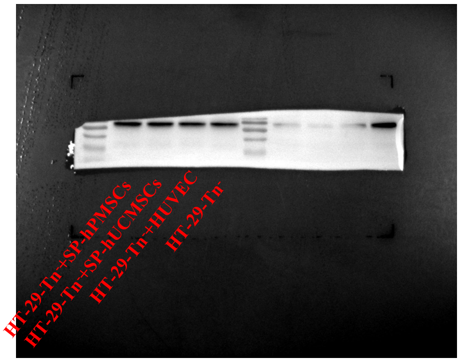


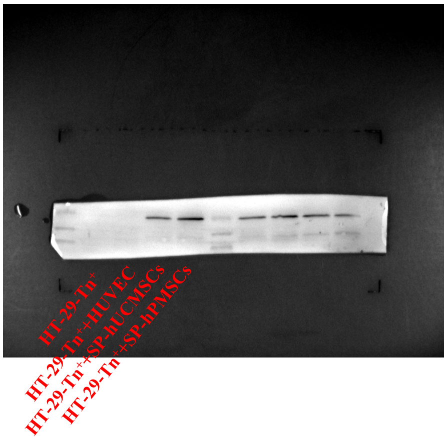

Supplement: Supplementary file 2 — Additional file 2: Three repeated Western blot experiments. [file 13287_2023_3334_MOESM2_ESM.pdf]
